# Supplementary material for: Environmental characteristics around the household and their association with hookworm infection in rural communities from Bahir Dar, Amhara Region, Ethiopia
Source: PLoS Negl Trop Dis. 2021 Jun 22;15(6):e0009466. doi: 10.1371/journal.pntd.0009466 (PMC8219153; doi:10.1371/journal.pntd.0009466)
Supplement: S1 Table — (DOCX) [file pntd.0009466.s001.docx]

### Environmental characteristics around the household and their association with hookworm infection in rural communities from Bahir Dar, Amhara Region, Ethiopia

Melaku Anegagrie^1,2,+^, Sofia Lanfri^3,4,+^, Aranzazu Amor Aramendia^1,2^, Carlos Matías Scavuzzo^3,5^, Zaida Herrador^2^, Agustín Benito^2^, Maria Victoria Periago^4*^.

1 Fundación Mundo Sano, Calle Recaredo 3, Madrid, 28002, Spain

2 National Centre for Tropical Medicine, Institute of Health Carlos III, Ctra. de Pozuelo 28, Majadahonda, Madrid, 28222, Spain

3 Instituto Gulich, CONAE, UNC, Ruta Provincial C45 a 8 Km, Falda del Cañete, Córdoba, (CP5187) Argentina

4 Fundación Mundo Sano, Paraguay 1535, Buenos Aires, (CP1061) Argentina

5 Consejo Nacional de Investigaciones Científicas y Técnicas (CONICET), Godoy Cruz 2290, Buenos Aires, (CP1425) Argentina

+ Authors contributed equally to this work.

*Corresponding author: María Victoria Periago, [vperiago@mundosano.org](mailto:vperiago@mundosano.org), Consejo Nacional de Investigaciones Científicas y Técnicas (CONICET), Fundación Mundo Sano, Paraguay 1535, Buenos Aires, (CP1061) Argentina.

**S1 Table**. Socioeconomic characteristics obtained using the extended questionnaire by village (Mazoria, Zenzelema and Sesaberet).

| \| **Socioeconomic characteristics** \| **Total**  **N = 138** \| **Mazoria**  **N = 39** \| **Sesaberet**  **N= 48** \| **Zenzelema**  **N= 41** \| \| --- \| --- \| --- \| --- \| --- \| \| Toilet distance : <=3 m  [No. (%)] \| 75 (56.0) \| 29 (74.4) \| 27 (57.0) \| 15 (36.6) \| \| Toilet distance: > 3 <= 10 m. [No. (%)] \| 59 (44.2) \| 9 (25.6) \| 21 (43.8) \| 26 (63.4) \| \| Share the Toilet: No.  [No. (%)] \| 27 (20.2) \| 7 (18.0) \| 38 (80.9) \| 35 (85.4) \| \| Share the Toilet: Yes  [No. (%)] \| 107 (79.9) \| 32 (82.1) \| 9 (19.2) \| 6 (14.6) \| \| Toilet type: latrine without slate. [No. (%)] \| 47 (34.1) \| 7 (18.0) \| 15 (31.3) \| 17 (41.5) \| \| Toilet type: open area/ bush. [No. (%)] \| 91 (65.9) \| 32 (82.1) \| 35 (68.8) \| 24 (58.5) \| \| Collect waste water: No  [No. (%)] \| 109 (81.3) \| 30 (76.9) \| 38 (80.9) \| 35 (85.4) \| \| Collect waste water: Yes  [No. (%)] \| 25 (18.7) \| 9 (23.1) \| 9 (19.2) \| 6 (14.6) \| \| Using excreta in agriculture: No. [No. (%)] \| 129 (97.0) \| 38 (100.0) \| 44 (93.6) \| 40 (97.6) \| \| Using excreta in agriculture: Yes. [No. (%)] \| 4 (3.0) \| 38 (100.0) \| 3 (6.4) \| 1 (2.4) \| \| Use of collected waste water: agriculture.  [No. (%)] \| 21 (87.5) \| 6 (66.7) \| 9 (100.0) \| 5 (100.0) \| \| Use of collected waste water: drinking water for animals. [No. (%)] \| 3 (12.5) \| 3 (33.3) \| No data \| No data \| \| Water source for bath: pipeline. [No. (%)] \| 12 (9.0) \| No data \| 11 (23.4) \| 1 (2.4) \| \| Water source for bath: river. [No. (%)] \| 39 (29.1) \| 31 (79.5) \| 7 (14.9) \| 1 (2.4) \| \| Water source for bath: treated boreholes.  [No. (%)] \| 14 (10.5) \| No data \| 14 (29.8) \| No data \| \| Water source for bath: well. [No. (%)] \| 69 (51.5) \| 8 (20.5) \| 15 (31.9) \| 39 (95.1) \| \| Water source for drinking during dry season: pipeline  [No. (%)] \| 27 (20.2) \| No data \| 21 (44.7) \| 6 (14.6) \| \| Water source for drinking during dry season: river.  [No. (%)] \| 21 (15.7) \| 9 (23.1) \| 3 (6.4) \| 9 (22.0) \| \| Water source for drinking during dry season: treated boreholes. [No. (%)] \| 14 (10.5) \| No data \| 14 (29.8) \| No data \| \| Water source for drinking during dry season: well. [No. (%)] \| 72 (53.7) \| 30 (76.9) \| 9 (19.2) \| 26 (63.4) \| \| Water source for drinking during rainy season: pipeline. [No. (%)] \| 17 (12.7) \| No data \| 13 (27.7) \| 4 (9.8) \| \| Water source for drinking during rainy season: river. [No. (%)] \| 38 (28.4) \| 28 (71.8) \| 8 (17.0) \| 2 (4.9) \| \| Water source for drinking during rainy season: treated boreholes.  [No. (%)] \| 15 (11.2) \| No data \| 15 (31.9) \| No data \| \| Water source for drinking during rainy season: well. [No. (%)] \| 62 (46.3) \| 11 (28.2) \| 9 (19.2) \| 35 (85.4) \| \| Water source for drinking during rainy season: other. [No. (%)] \| 2 (1.5) \| No data \| 2 (4.3) \| No data \| \| Water source for cooking: pipeline. [No. (%)] \| 18 (13.4) \| No data \| 16 (34.0) \| 2 (4.9) \| \| Water source for cooking: river. [No. (%)] \| 30 (22.4) \| 25 (64.1) \| 4 (8.5) \| 1 (2.4) \| \| Water source for cooking: treated boreholes.  [No. (%)] \| 15 (11.2) \| No data \| 15 (31.9) \| No data \| \| Water source for cooking: well. [No. (%)] \| 71 (53.0) \| 14 (35.9) \| 12 (25.5) \| 38 (92.7) \| \| Water source for handwashing: pipeline. [No. (%)] \| (12.9) \| No data \| 14 (30.4) \| 3 (7.3) \| \| Water source for handwashing: river.  [No. (%)] \| 34 (25.8) \| 27 (71.1) \| 6 (13.0) \| 1 (2.4) \| \| Water source for handwashing: treated boreholes. [No. (%)] \| 13 (9.9) \| No data \| 13 (28.3) \| No data \| \| Water source for handwashing: well.  [No. (%)] \| 68 (51.5) \| 11 (29.0) \| 13 (28.3) \| 37 (90.2) \| \| Presence of domestic animals: No. [No. (%)] \| 57 (41.6) \| 11 (29.0) \| 22 (46.8) \| 21 (51.2) \| \| Presence of domestic animals: Yes. [No. (%)] \| 80 (58.4) \| 28 (71.8) \| 25 (53.2) \| 20 (48.8) \| \| Electricity presence: No  [No. (%)] \| 97 (70.3) \| 39 (100.0) \| 46 (95.8) \| 5 (12.2) \| \| Electricity presence: Yes  [No. (%)] \| 41 (29.7) \| No data \| 2 (4.2) \| 36 (87.8) \| \| Religion: Muslim. [No. (%)] \| 4 (2.9) \| 2 (5.3) \| 1(2.1) \| No data \| \| Religion: Orthodox.  [No. (%)] \| 132 (97.1) \| 36 (94.7) \| 46 (97.9) \| 41 (100.0) \| \| Sex of head of household: female. [No. (%)] \| 17 (12.9) \| 5 (13.9) \| 5 (10.9) \| 7 (17.5) \| \| Sex of head of household: male. [No. (%)] \| 115 (87.1) \| 31 (86.1) \| 41 (89.1) \| 33 (82.5) \| \| Members with monthly income : 1. [No. (%)] \| 103 (79.8) \| 26 (72.2) \| 33 (78.6) \| 34 (82.9) \| \| Members with monthly income : 2, 3 or 5.  [No. (%)] \| 26 (20.2) \| 10 (27.8) \| 9 (21.43) \| 7 (17.1) \| \| Number of individuals under 5 years of age: 0  [No. (%)] \| 58 (47.2) \| 19 (50.0) \| 21 (44.7) \| 15 (53.6) \| \| Number of individuals under 5 years of age: 1  [No. (%)] \| 57 (46.3) \| 17 (44.7) \| 26 (55.32) \| 8 (28.57) \| \| Number of individuals under 5 years of age: 2  [No. (%)] \| 8 (6.5) \| 2 (5.3) \| No data \| 5 (17.9) \| \| Number of individuals older than 5: from 1 to 5  [No. (%)] \| 105 (77.8) \| 26 (58.4) \| 44 (80.9) \| 33 (80.5) \| \| Number of individuals older than 5: from 6 to 9  [No. (%)] \| 30 (22.2) \| 12 (31.9) \| 9 (19.2) \| 8 (19.5) \| \| Own the land: No.  [No. (%)] \| 28 (20.7) \| 4 (10.3) \| 5 (10.4) \| 19 (46.3) \| \| Own the land: Yes  [No. (%)] \| 107 (79.3) \| 35 (89.7) \| 43 (89.6) \| 22 (53.7) \| \| Radio presence: No.  [No. (%)] \| 103 (75.7) \| 33 (84.6) \| 38 (80.9) \| 24 (60.0) \| \| Radio presence: Yes  [No. (%)] \| 33 (24.3) \| 6 (15.4) \| 9 (19.2) \| 16 (40.0) \| \| Head Main occupation: farmer. [No. (%)] \| 87 (71.9) \| 19 (63.3) \| 26 (65.0) \| 36 (87.8) \| \| Head Main occupation: laborer. [No. (%)] \| 15 (12.4) \| 4 (13.3) \| 7 (17.5) \| 2 (4.9) \| \| Head Main occupation: trader. [No. (%)] \| 13 (10.7) \| 5 (16.7) \| 4 (10.0) \| 2 (4.9) \| \| Head Main occupation: other. [No. (%)] \| 5 (4.1) \| 1 (3.3) \| 3 (7.5) \| 1 (2.4) \| \| Mean age of household head. (mean) \| 43.0 \| 45.0 \| 42.0 \| 42.0 \| \| Mean number of years that household head attended school. (mean) \| 6.0 \| 6.0 \| 6.0 \| No data \|   *Note: Socioeconomic characteristics. |
| --- | --- | --- | --- | --- | --- | --- | --- | --- | --- | --- | --- | --- | --- | --- | --- | --- | --- | --- | --- | --- | --- | --- | --- | --- | --- | --- | --- | --- | --- | --- | --- | --- | --- | --- | --- | --- | --- | --- | --- | --- | --- | --- | --- | --- | --- | --- | --- | --- | --- | --- | --- | --- | --- | --- | --- | --- | --- | --- | --- | --- | --- | --- | --- | --- | --- | --- | --- | --- | --- | --- | --- | --- | --- | --- | --- | --- | --- | --- | --- | --- | --- | --- | --- | --- | --- | --- | --- | --- | --- | --- | --- | --- | --- | --- | --- | --- | --- | --- | --- | --- | --- | --- | --- | --- | --- | --- | --- | --- | --- | --- | --- | --- | --- | --- | --- | --- | --- | --- | --- | --- | --- | --- | --- | --- | --- | --- | --- | --- | --- | --- | --- | --- | --- | --- | --- | --- | --- | --- | --- | --- | --- | --- | --- | --- | --- | --- | --- | --- | --- | --- | --- | --- | --- | --- | --- | --- | --- | --- | --- | --- | --- | --- | --- | --- | --- | --- | --- | --- | --- | --- | --- | --- | --- | --- | --- | --- | --- | --- | --- | --- | --- | --- | --- | --- | --- | --- | --- | --- | --- | --- | --- | --- | --- | --- | --- | --- | --- | --- | --- | --- | --- | --- | --- | --- | --- | --- | --- | --- | --- | --- | --- | --- | --- | --- | --- | --- | --- | --- | --- | --- | --- | --- | --- | --- | --- | --- | --- | --- | --- | --- | --- | --- | --- | --- | --- | --- | --- | --- | --- | --- | --- | --- | --- | --- | --- | --- | --- | --- | --- | --- | --- | --- | --- | --- | --- | --- | --- | --- | --- | --- | --- | --- | --- | --- | --- | --- | --- | --- | --- | --- | --- | --- | --- | --- | --- | --- | --- | --- | --- | --- | --- | --- | --- | --- | --- | --- | --- | --- | --- | --- | --- | --- | --- | --- | --- |
