# Supplementary material for: Environmental characteristics around the household and their association with hookworm infection in rural communities from Bahir Dar, Amhara Region, Ethiopia
Source: PLoS Negl Trop Dis. 2021 Jun 22;15(6):e0009466. doi: 10.1371/journal.pntd.0009466 (PMC8219153; doi:10.1371/journal.pntd.0009466)
Supplement: Attachment — Submitted filename: PNTD-D-21-00055_reviewer comments.pdf [file pntd.0009466.s003.pdf]

PLOS Neglected Tropical Diseases

Environmental characteristics around the household and their association with hookworm infection in rural communities from Bahir Dar, Amhara Region, Ethiopia

--Manuscript Draft--

**Manuscript Number:**	PNTD-D-21-00055
**Full Title:**	Environmental characteristics around the household and their association with hookworm infection in rural communities from Bahir Dar, Amhara Region, Ethiopia
**Short Title:**	Hookworm infection and environmental characteristics around households
**Article Type:**	Research Article
**Keywords:**	Soil-transmitted helminths, hookworm, geographic information systems, remote sensing, environmental characteristics
**Abstract:**	Soil-Transmitted Helminths (STH) are highly prevalent Neglected Tropical Disease in Ethiopia, an estimated 26 million are infected. Geographic Information Systems and Remote Sensing (RS) technologies assist data mapping and analysis, and the prediction of the spatial distribution of infection in relation to environmental variables. The influence of socioeconomic, environmental and soil characteristics on hookworm infection at the individual and household level is explored in order to identify spatial patterns of infection in rural villages from Zenzelema (Amhara region). Inhabitants greater than 5 years old were recruited in order to assess the presence of STH. Socioeconomic and hookworm infection variables at the household level and environmental variables and soil characteristics using RS were obtained. The dominant STH found was hookworm. Individuals which practiced open defecation and those without electricity had a significant higher number of hookworm eggs in their stool. Additionally, adults showed statistically higher hookworm egg counts than children. Nonetheless, the probability of hookworm infection was not determined by socioeconomic conditions but by environmental characteristics surrounding the households, including a combination of vigorous vegetation and bare soil, high temperatures, and compacted soils (high bulk density) with more acidic pH. The identification of high-risk environmental areas provides a useful tool for planning, targeting and monitoring of control measures, including not only children but also adults when hookworm is concerned.
**Additional Information:**	
**Question**	**Response**
**Financial Disclosure**<br><br>Enter a financial disclosure statement that describes the sources of funding for the work included in this submission. Review the submission guidelines for detailed requirements. View published research articles from *PLOS NTDs* for specific examples.<br><br>This statement is required for submission and **will appear in the published article** if the submission is accepted. Please make sure it is accurate.	The authors received no specific funding for this work.

Powered by Editorial Manager® and ProduXion Manager® from Aries Systems Corporation

**Unfunded studies**<br><br>Enter: *The author(s) received no specific funding for this work.*<br><br>**Funded studies**<br><br>Enter a statement with the following details:<br>• Initials of the authors who received each award<br>• Grant numbers awarded to each author<br>• The full name of each funder<br>• URL of each funder website<br>• Did the sponsors or funders play any role in the study design, data collection and analysis, decision to publish, or preparation of the manuscript?<br>• **NO** - Include this sentence at the end of your statement: *The funders had no role in study design, data collection and analysis, decision to publish, or preparation of the manuscript.*<br>• **YES** - Specify the role(s) played.<br><br>* typeset	
**Competing Interests**<br><br>Use the instructions below to enter a competing interest statement for this submission. On behalf of all authors, disclose any competing interests that could be perceived to bias this work—acknowledging all financial support and any other relevant financial or non-financial competing interests.<br><br>This statement **will appear in the published article** if the submission is accepted. Please make sure it is accurate. View published research articles from *PLOS NTDs* for specific examples.	The authors have declared that no competing interests exist.

Powered by Editorial Manager® and ProduXion Manager® from Aries Systems Corporation

**NO authors have competing interests**<br><br>Enter: *The authors have declared that no competing interests exist.*<br><br>**Authors with competing interests**<br><br>Enter competing interest details beginning with this statement:<br><br>*I have read the journal's policy and the authors of this manuscript have the following competing interests: [insert competing interests here]*<br><br>* typeset<br><br>This statement is required for submission and **will appear in the published article** if the submission is accepted. Please make sure it is accurate and that any funding sources listed in your Funding Information later in the submission form are also declared in your Financial Disclosure statement.	
**Data Availability**<br><br>Authors are required to make all data underlying the findings described fully available, without restriction, and from the time of publication. PLOS allows rare exceptions to address legal and ethical concerns. See the PLOS Data Policy and FAQ for detailed information.	Yes - all data are fully available without restriction

Powered by Editorial Manager® and ProduXion Manager® from Aries Systems Corporation

A Data Availability Statement describing where the data can be found is required at submission. Your answers to this question constitute the Data Availability Statement and **will be published in the article**, if accepted.<br><br>**Important:** Stating 'data available on request from the author' is not sufficient. If your data are only available upon request, select 'No' for the first question and explain your exceptional situation in the text box.<br><br>Do the authors confirm that all data underlying the findings described in their manuscript are fully available without restriction?	
**Describe where the data may be found in full sentences. If you are copying our sample text, replace any instances of XXX with the appropriate details.**<br><br>• If the data are **held or will be held in a public repository**, include URLs, accession numbers or DOIs. If this information will only be available after acceptance, indicate this by ticking the box below. For example: *All XXX files are available from the XXX database (accession number(s) XXX, XXX.*).<br>• If the data are all contained **within the manuscript and/or Supporting Information files**, enter the following: *All relevant data are within the manuscript and its Supporting Information files.*<br>• If neither of these applies but you are able to provide **details of access elsewhere**, with or without limitations, please do so. For example:<br><br>*Data cannot be shared publicly because*	All relevant data are within the manuscript and its Supporting Information files.

Powered by Editorial Manager® and ProduXion Manager® from Aries Systems Corporation

of [XXX]. Data are available from the XXX Institutional Data Access / Ethics Committee (contact via XXX) for researchers who meet the criteria for access to confidential data.<br><br>*The data underlying the results presented in the study are available from (include the name of the third party and contact information or URL).*<br>• This text is appropriate if the data are owned by a third party and authors do not have permission to share the data.<br><br>* typeset	
Additional data availability information:	Tick here if the URLs/accession numbers/DOIs will be available only after acceptance of the manuscript for publication so that we can ensure their inclusion before publication.

Manuscript

Click here to access/download;Manuscript;STH Manuscript PLoS.docx

Title

Environmental characteristics around the household and their association with hookworm infection in rural communities from Bahir Dar, Amhara Region, Ethiopia

Short Title

Hookworm infection and environmental characteristics around households

Authors and affilitations

Melaku Anegagrie[1,2,+], Sofia Lanfri[3,4,+], Aranzazu Amor Aramendia[1,2], Carlos Matías Scavuzzo[3,5], Zaida Herrador[2], Agustín Benito[2], Maria Victoria Periago[4*].

1 Fundación Mundo Sano, Calle Recaredo 3, Madrid, 28002, Spain

2 National Centre for Tropical Medicine, Institute of Health Carlos III, Ctra. de Pozuelo 28, Majadahonda, Madrid, 28222, Spain

3 Instituto Gulich, CONAE, UNC, Ruta Provincial C45 a 8 Km, Falda del Cañete, Córdoba, (CP5187) Argentina

4 Fundación Mundo Sano, Paraguay 1535, Buenos Aires, (CP1061) Argentina

5 Consejo Nacional de Investigaciones Científicas y Técnicas (CONICET), Godoy Cruz 2290, Buenos Aires, (CP1425) Argentina

+ Authors contributed equally to this work.

*Corresponding author: María Victoria Periago, vperiago@mundosano.org, Consejo Nacional de Investigaciones Científicas y Técnicas (CONICET), Fundación Mundo Sano, Paraguay 1535, Buenos Aires, (CP1061) Argentina.

Abstract

Soil-Transmitted Helminths (STH) are highly prevalent Neglected Tropical Disease in Ethiopia, an estimated 26 million are infected. Geographic Information Systems and Remote Sensing (RS) technologies assist data mapping and analysis, and the prediction of the spatial distribution of infection in relation to environmental variables. The influence of socioeconomic, environmental and soil characteristics on hookworm infection at the individual and household level is explored in order to identify spatial patterns of infection in rural villages from Zenzelema (Amhara region). Inhabitants greater than 5 years old were recruited in order to assess the presence of STH. Socioeconomic and hookworm infection variables at the household level and environmental variables and soil characteristics using RS were obtained. The dominant STH found was hookworm. Individuals which practiced open defecation and those without electricity had a significant higher number of hookworm eggs in their stool. Additionally, adults showed statistically higher hookworm egg counts than children. Nonetheless, the probability of hookworm infection was not determined by socioeconomic conditions but by environmental characteristics surrounding the households, including a combination of vigorous vegetation and bare soil, high temperatures, and compacted soils (high bulk density) with more acidic pH. The identification of high-risk environmental areas provides a useful tool for planning, targeting and monitoring of control measures, including not only children but also adults when hookworm is concerned.

Author Summary

Soil-Transmitted Helminths (STH) are a group of intestinal parasites that are included in the list of Neglected Tropical Diseases (NTDs) elaborated by the World Health Organization (WHO). This group includes roundworms, whipworms, and hookworms. Sub-Saharan Africa (SSA) is one of the most largely affected by NTDs and Ethiopia harbours one of the largest burdens of STH, especially hookworm, with 10 million infected. In this study we aimed to explore the association between the environment, soil and socioeconomic characteristics most associated with the presence of hookworm infection in a rural area from Bahir Dar, Amhara Region, Ethiopia. Results of this study showed that the presence of hookworm around the household is associated to environmental characteristics such as high temperatures, a

combination of vigorous vegetation and bare compacted soil and acidic pH. On the other hand, the intensity of hookworm infection was associated to certain socioeconomic conditions such as the lack of latrines with the practice of open defecation and a lack of electricity. Therefore, in order for the infection to establish itself in a community, certain environmental characteristics are needed, but once the infection is established, certain socioeconomic characteristics play a role in its transmission pattern.

Keywords

Soil-transmitted helminths, hookworm, geographic information systems, remote sensing, environmental characteristics

Introduction

Neglected Tropical Diseases (NTDs) are widespread in Sub-Saharan African (SSA) countries with an estimated 500 million people being affected by the most common NTDs, including Soil-Transmitted Helminthiases, Schistosomiasis, Lymphatic Filariasis, Trachoma and Onchocerciasis (1). Ethiopia is not an exception and it is one of the countries with the highest burden of disease in SSA, with an estimated 80 million people living in NTD endemic areas out of a population of 115 million (2); with the presence of almost all 20 diseases on the list, with very few exceptions (3). The Soil-Transmitted Helminths (STH) are the most prevalent in the country, with an estimated 10 million people infected with hookworm, 26 million with *Ascaris lumbricoides* and 21 with *Trichuris trichiura* (1). Moreover, the presence of *Strongyloides stercoralis* has also been detected (4-7), but the actual burden is unknown since the techniques usually used for diagnosis of STH are not sensitive enough for this parasite (5).

In 2004, Ethiopia launched a deworming strategy focused on preschool aged children (PSAC) that was implemented in every district of the country, except the capital city of Addis Ababa (3). The program was organized by the Regional Health Bureaus (RHB) and by 2009; it had reached 11 million children (3). In 2013, the Federal Democratic Republic of Ethiopia Ministry of Health (FMHO) launched its first NTD Master Plan and established a NTD Case Team within the Ministry (8). National guidelines for STH and

Schistosoma were put in place in 2014 (9) and in 2015 a national deworming program for school aged children (SAC) was launched (10), with a revision and update of the NTD Master Plan in 2016 (11). The number of people living in endemic areas for STH was approximately 79 million and the population living in areas that would qualify for mass drug administration (MDA) is comprised of 4.6 million PSAC, 17.7 million SAC and 31.3 million adults (10, 11). A total of 476 woredas were identified through these different studies as in need of MDA for STH with prevalences ranging from 20% to greater than 50%, so that 279 woredas would require treatment twice per year according to World Health Organization (WHO) guidelines (10). The regional states most affected were Amhara, Gambela, Southern Nations, Nationalities and Peoples (SNNP) and the western part of Oromia.

Understanding the distribution of STH in specific areas as well as the factors most associated with their presence would allow tailoring programs to not only identify those areas most likely to be affected for allocation of resources but also to promote health practices to avoid reinfection after treatment. Geographic Information Systems (GIS) and Remote Sensing (RS) technologies assist not only data mapping and analysis but also the prediction of the spatial distribution of infection in relation to RS and environmental variables (12). Maps specific for STH have been developed in different countries in order to determine areas at risk for the presence of these parasites, most of them using historical prevalence data from either point studies (13-16) or national STH data (17), but none of these were conducted at the household level. Most studies conducted at the household level have determined certain risk factors for infection with STH, but environmental parameters were not included (18-21).

Previous studies conducted in Amhara have shown that hookworm is the predominant species of STH present in rural areas (4-6, 22-24). Given that Ethiopia is the country with third highest burden of hookworm in SSA, in the current study we explore the influence of socioeconomic, environmental and soil characteristics on the infection of hookworm at the individual and household level in order to identify spatial patterns of infection in a community from a rural *kebele* of Amhara region, Ethiopia. Highlighting

the identification of high-risk areas could provide a very useful tool for planning, targeting and monitoring of control measures.

Methods

Study area. Ethiopia is a Federal Democratic Republic comprised of ten regional states and two administrative cities which are then subdivided into 95 zones and 839 administrative *woredas* or districts. *Woredas* are further divided into 16,253 *kebeles*, the smallest administrative unit. Most of the country is suitable for the transmission of these parasites. The current study was conducted in the Amhara Region which is located in the north-western part of the country. The study was carried out in the rural *kebele* of Zenzelema, about 20 km east of Bahir Dar City, the capital of Amhara, in the South shore of Tana Lake (Fig. 1). The district is located in the highlands, at 1,700 to 2,000 m above sea level. The area is under the influence of three seasons: rainy, from June to September; spring, from October until January; and a dry season the rest of the year. This *kebele* is made of nine *gotts* or villages; five of them were randomly selected for the study (6).

Zenzelema, located in the main road, with poor access to water, Mazoria and Sesaberet which are accessible by walking through earth tracks and have smaller streams and watercourses with natural water, and Kurbi and Gedro, located in more remote areas near Tana Lake, with no access tracks. In all these villages, except for Zenzelema, the houses are far apart from one another and surrounded by crops and wooded areas. On the other hand, Zenzelema is a congested settlement where houses are adjacent to one another (Fig. 2). The family subsistence economy is based on small cultivations and livestock (mainly cattle, sheep and goats). Even though the area is on the shore of Blue Nile and Tana Lake, water resources for cultivation are markedly dependent on the rain, with no existing infrastructure for rainwater collection. Moreover, land degradation because of erosion, mostly related to high deforestation rates, is a big concern, with high amounts of soil lost every year.

Data collection

The data of this study were collected in the frame of a larger research aimed at determining the prevalence of the STH *S. stercoralis* at the school and community level in an area known to be of high

prevalence for hookworm (5, 6). Briefly, all inhabitants over 5 years of age, living in the area for at least three months, were invited to participate. Information about individual (children and adult) and household characteristics was obtained by using WHO standardized surveys, adapted to the Ethiopian culture and translated into Amharic language. All participants were asked to answer an individual questionnaire while the head of the family was also asked to answer a household questionnaire. From February to June 2016, a field team composed of a nurse, two HEW and the project coordinator went house to house, to collect the samples, conduct the questionnaires and georeference the households. Stool samples were processed as previously described (6) to determine presence and intensity of hookworm infection.

Household characterization. Through the different questionnaires and sample collection, socioeconomic and hookworm infection variables were obtained and analysed at the household level using a geographic information system (QGIS software). Several socioeconomic variables were measured related to the number of individuals per household, income, toilet type, water source, presence of electricity, etc. These are further detailed in the result section. The parasitological parameters used for hookworm were the number of hookworm infected individuals per household, and the mean number of EPG per household (mean EPG of infected individuals per household).

Soil and Environmental characterization. The environmental variables selected for analysis were those related to the variables reported as significant in previous studies that focused on the risk of soil-transmitted helminth infections (13-17, 20, 38(25), 44-49(26-31)). The selected environmental variables obtained through remote sensors were precipitation, temperature, NDVI, NDBI, MSI and BSI. These are listed in Table 1, together with their source, characteristics and indexes used to calculate them.

Table 1. Environmental variables used with their characteristics, sources and indexes used to calculate them.

Environmental Variables	Source	Characteristics/ Sentinel-2A Indexes

Precipitation	WorldClim version 2.1 climate data for 1979-2000 (New version released in January 2020)	Spatial resolution of 30 seconds (~1 km2)/ NA
Temperature	WorldClim version 2.1 climate data for 1979-2000 (New version released in January 2020)	Mean temperature from monthly January/ NA
Normalized Difference Vegetation Index (NDVI)	Satellite imagery Sentinel-2A of 01 Feb 2017*<br><br>Level 1C product	Numerical indicator highly associated with vegetation content/ (B8 – B4) / (B8 + B4)
Normalized Difference Built-up Index (NDBI)	Satellite imagery Sentinel-2A of 01 Feb 2017*<br><br>Level 1C product	Highlights urban areas/ (B11-B8)/(B11+B8)
Moisture Stress Index (MSI)	Satellite imagery Sentinel-2A of 01 Feb 2017*<br><br>Level 1C product	Index of hydric stress/ (B11 / B08)
Bare Soil Index (BSI)	Satellite imagery Sentinel-2A of 01 Feb 2017*<br><br>Level 1C product	Numeric indicador that enhances the identification of bare soil areas and fallow lands/ (B11 + B4) – (B8 + B2) / (B11 + B4) + (B8 + B2)

*Note: Source of ESA, image courtesy of the U.S. Geological Survey. Free Sentinel-2A imagery was downloaded from the ESA Sentinel data hub (https://scihub.copernicus.eu).

With respect to the interpretation of these indexes, high NDVI values correspond to areas that reflect more in the near-infrared spectrum, which indicates denser and healthier vegetation (50(32)). This index is related to MSI since it is a measure of hydric stress, with the highest numbers indicating greater hydric

stress of plants and therefore a lower content of soil humidity (51(33)). The values for MSI range from 0 to greater than 3, so the typical range of this index for vegetation is around 0.2 to 2 (52(34)). On the other hand, NDBI highlights urban areas and is very helpful for estimating surfaces with buildings with respect to bare areas or those with vegetation (53(35)). Finally, BSI is related to NDBI in the sense that it is a numeric indicator that enhances the identification of bare soil areas and fallow lands (54(36)).

Soil variables with greater spatial resolution (250 m) were available for Ethiopia. Those variables related to the ones previously used (13); organic carbon content, acidity, bulk density and gypsum content were chosen and are listed in Table 2. BLDFIE is the ratio of the oven-dry mass of the solids to the volume of the soil; the bulk volume includes not only the volume of the solids but also that of the pore space (55(37)). With respect to the pH variables of the soil, they describe more than relative acidity or alkalinity, it also provides information on nutrient availability, metal dissolution chemistry, and the activity of microorganisms (56(38)), and therefore it might influence the presence or absence of hookworm larvae in the soil. The remaining soil variables refer to the depth of the bedrock (BDRICM) in cm to a lithic or paralithic contact and its composition, with the weight percentage of the clay particles (CLYPPT) as < 2 µm of the mass fraction of soil material at < 2 mm, and the volumetric percentage of coarse fragments (CRFVOL) as the mass fraction of the soil material at > 2 mm (55(37)).

Table 2. Soil variables used with their abbreviation, characteristics and unit of measurement.

**Soil variables**	**Abbreviation**	**Characteristics (Unit)**
Soil organic carbon content	ORCDRC	NA (permille)
Soil organic carbon stock	Ocstha	NA (ton/ha)
Bulk density	BLDFIE	fine earth (kg/m3)
pH index measured in KCl solution	Phikcl	Relative acidity or alkalinity
pH index measured in water	Phihox	Relative acidity or alkalinity

solution		
Weight percentage of the silt particles	SLTppt	Particles about 0.0002–0.05 mm (percentage)
Cation Exchange Capacity of soil	CECSOL	NA (cmolc/kg)
Depth to bedrock	BDRICM	R horizon up to 200 cm (cm)
Weight percentage of the clay particles.	CLYPPT	Particles about <0.0002 mm (percentage)
Volumetric percentage of coarse fragments.	CRFVOL	Coarse fragments about >2 mm (percentage)

*Note: NA: Not Applicable. Source: ISRIC — World Soil Information (37).

In order to determine any association with hookworm infection between any of these environmental and soil variables, given the route of infection of the parasite with a necessary passage through the soil, the average of each of these variables were estimated with a 30 m buffer radius around each household using QGIS software.

Statistical Analysis. SataScan was used to analyse the geographic spatial pattern of the parasitological indexes in the study area (57, 58(39, 40)). The presence of statistically significant spatial conglomerates was analysed, as well as that of the non-random distribution in space. This was performed by gradually scanning a window across space, noting the number of observed and expected observations inside the window at each location, and a p-value was assigned to the cluster. Scan statistics use a different probability model depending on the nature of the data. In this study, a Purely Spatial analysis was performed, with the objective of scanning for clusters with high values using the normal model for continuous data (58(40)). The unit of analysis was the household and the variables evaluated were mean EPG per household and number of individuals infected.

The association between individual EPG and characteristics of the household was analysed using the Mann-Whitney U (MWU) and Kruskal-Wallis H (KWH) tests. Chi square test was used for comparing the prevalence of the infection between villages. The individual social and economic characteristics evaluated are listed in the results section. Estimation of the household risk of hookworm infection was performed through a linear multiple regression analysis. The number of hookworm infected individuals per household as the dependent variable was modelled in relation to the predictive variables: environmental (Table 1), soil characteristics (Table 2) and socioeconomic variables (Supplementary Table 1). Statsmodel Python module (statsmodel.org) was used for the comparison between groups as well as to adjust the regression model.

Ethics approval and consent to participate

The Amhara National Regional State Health Bureau Ethics Review Committee revised and approved the study protocol (Ref. nº: 1/87/2008). According to the principles of the Helsinki Declaration, informed consent for stool examination was sought, as well as withdrawal, guarantee of anonymity, treatment and follow up. Written informed consent was set for adults and children; for the second group, parents or guardians signed the consent form.

Results

Hookworm infection characteristics and spatial distribution. A total of 792 individuals from 241 households participated in the study by providing stool samples: 152 individuals from Mazoria, 193 from Sesaberet, 160 from Gedro, 139 from Kurbi and 148 from Zenzelema. The descriptive characteristics of hookworm infection in the five villages are detailed in Table 4. The mean number of people infected with hookworm was lower in Zenzelema (64.2%) and highest in Kurbi (87.8%), although the difference in prevalence and the mean eggs per gram of feces (EPG) per household between the villages was not statistically significant. In the larger previous study conducted in this study population (6), the presence of all five species of STH was detected, with a dominance of hookworm in Mazoria and Sesaberet, and a dominance of *S. stercoralis* in Zenzelema. With respect to hookworm, Kurbi not only had the highest

prevalence of infection (87.8%) but also the greatest average of infected individuals per household (3.0). Nonetheless, the highest mean EPG per household was observed in Gedro (1442.7 EPG). With respect to the intensity of hookworm infection, most infections were light and the only heavy intensity infection was found in Gedro (Table 4).

Table 4. Infection status of the participants of the study from the five villages, Kurbi, Gedro, Mazoria, Sesaberet and Zenzelema (Amhara State, Ethiopia).

**Hookworm infection characteristics**	**Mazoria**<br>**N = 152**	**Sesaberet**<br>**N = 193**	**Gedro**<br>**N = 160**	**Kurbi**<br>**N = 139**	**Zenzelema**<br>**N = 148**
Prevalence [%]	79.6	78.2	83.8	87.8	64.2
Number of infected individuals [No.]	121	151	134	122	95
No. of infected individuals by intensity as per MM [No.]	121:<br>120 light<br>4 moderate	150*:<br>144 light<br>6 moderate	134:<br>129 light<br>4 moderate<br>1 heavy	122:<br>113 light<br>9 moderate	94*:<br>144 light<br>6 moderate
Mean number of infected individuals per household (mean)	2.5	2.7	2.9	3.0	2.3
Mean EPG per	1005.1	1002.3	1442.7	1273.4	1224.5

household (mean)					

*Note: MM: MacMaster Technique. EPG: Eggs per gram of feces. Intensity of infection for hookworm was measured according to WHO guidelines where: 1 to 1999 EPG is a light infection, 2000 to 3999 EPG is medium and >4000 EPG is heavy (59(41)). *Lack of MM data for one sample from Sesaberet and one sample from Zenzelema.

As far as the hookworm infection per household, the spatial distribution among the five villages is shown in Figure 3. In this figure, the data listed in Table 4 is verified. Most of the households presented at least three infected individuals (Fig. 3A) and the burden of infection was low, with more than 80% of the participants harbouring light hookworm infections and very few households with individuals harbouring moderate or high intensity infections (Fig. 3B). Kurbi, Gedro and Sesaberet had one household each with moderate mean EPG for the entire household. On the other hand, none of the villages contained households with high intensity mean EPG. Moreover, in Zenzelema, none of the households had more than five infected individuals and all of the average hookworm infections were of light intensity. According to the spatial cluster analysis, using the normal Statscan model, two clusters with high values for mean EPG were found in Gedro and Zenzelema while high value clusters for the number of infected individuals were found in Mazoria, Gedro and Kurbi. The spatial localization of these clusters is observed in Figure 4. In Gedro, the cluster for the number of infected individuals partially coincided with the cluster with high values of mean EPG.

The spatial distribution found was in agreement with what was observed in the terrain, with clear differences between the villages, as already described in the method section, but especially between Zenzelema and the rest of the villages. The habitat surrounding the houses was clearly different and more appropriate for the development of larvae in Mazoria than in Zenzelema as can be observed in Figure 3. The area around the households in Mazoria was more densely vegetated, with covered soil that tends to retain certain moisture and shade, versus the surroundings in Zenzelema where the houses are adjacent to each other and the area around the houses was more urbanized. In an area like Mazoria or Gedro,

more fields for cultivation were observed serving as cover for the practice of open defecation than in a setting like Zenzelema.

Household Socioeconomic characterization. The database generated consisted of 138 households with complete socioeconomic characterization corresponding to the villages of Sesaberet, Mazoria and Zenzelema. The average number of inhabitants per household was four. These 138 households were all the same with respect to the roof, floor and wall characteristics. All the roofs were made of corrugated iron, all the floors were made of straw pasted with mud/cow dung and all the walls were made of wood and mud with straw. Supplementary Table 1 describes the socioeconomic variables collected for each household. A total of 212 households participated in the simple household questionnaire and a sub-group of 138 household participated in the extended questionnaire which included individual information on the head of the household. The relation between the level of EPG with respect to all the measured socioeconomic variables was explored. Figure 5 shows the graphical representation using box plots only of those variables which showed statistical significance with respect to the level of EPG observed ($p<0.05$).

As observed in Figure 5, those individuals which practiced open defecation had a significant higher number of hookworm eggs in their stool. This was also true for those that did not have electricity, and those that used water from treated boreholes for cooking and handwashing. Moreover, adults showed statistically significant higher hookworm egg counts than children. Adults that own their own land also had significant higher eggs counts.

Spatial distribution of environmental and soil characteristics. The spatial distribution of some of the analysed soil and environmental indexes evaluated in the regions comprising all five villages is shown in Figure 6. The different characteristics were quite uniform between the villages, including altitude, although the area between Kurbi and Gedro has higher vegetation and therefore the values for the normalized difference vegetation index (NDVI) were high, while the values for the moisture stress index (MSI), bare soil index (BSI) and normalized difference built-up index (NDBI) were low. This makes sense since NDVI and MSI are associated with vegetation content and hydric stress while BSI and NDBI are

associated with urban areas and bare soil or fallow lands. Taking a closer look by village, Gedro, Sesaberet and Kurbi had the highest average values for NDVI around the households (0.31, 0.27 and 0.27 respectively), while the lower average values were found in Zenzelema (0.23) and Mazoria (0.23), while both Gedro and Zenzelema had higher average values of MSI around the households (1 and 0.93, respectively). Mazoria also had a high average value of BSI around the households (-0.017), while both Gedro and Zenzelema had low values (-0.35 and -0.33, respectively). The average NDBI index was higher in Zenzelema (-0.008) and Mazoria (-0.004) with respect to the rest of the villages (Gedro -0.035, Kurbi -0.023 and Sesaberet -0.022) and the average Dry bulk density index (BLDFIE)was highest in Sesaberet (1359.83) and lowest in Zenzelema (1320.18).

Taking the different parameters together, along with the infection characteristics, Kurbi had a high cluster of infected individuals in households that are located in an area with a high vegetation and hydric stress index. Mazoria also had a high cluster of infected individuals in households that were closer to an area with vegetation, higher hydric stress but also some bare soil. On the other hand, Gedro and Sesaberet had higher vegetation cover, higher bulk density and lower hydric stress and construction index, although Gedro had a high cluster of infected people while Sesaberet did not. Gedro also had a high cluster of households with high mean EPG and both of these clusters were located in households that were closer to an area with more bare soil. The households in Zenzelema presented surroundings with lower vegetation cover, lower proportion of bare soil and lower bulk density with higher hydric stress and construction index (BSI), and it also presented a high cluster of mean EPG per household. Sesaberet was the only village with no clusters of infection or high mean EPG and the households were surrounded by more vegetation and higher bulk density.

Household risk of hookworm infection. Only 89 households had complete socioeconomic data (all variables in the questionnaire were obtained). These households correspond to the villages of Sesaberet (SE), Mazoria (MA) and Zenzelema (ZE), thus including 368 individuals. This smaller database was used to model the risk of infection (number of individuals infected by hookworm per household – Ind_Numb). The model obtained through the linear multiple regression analysis showed that only the following

variables significantly contributed to the model ($p<0.05$): BSI, MSI, Ind_Numb, and Soil organic carbon concentration (ORCDRC) (R^2: 0.756, AIC: 229.4, the coefficients were 3.4041, -3.7972, 7.7182, and 1.1548 respectively). Given only environmental characteristics and none of the socioeconomic characteristics from the extended questionnaire significantly contributed to the model, all the households with simplified questionnaires were included in order to broaden the database and explore the risk of infection in the entire study area. Accordingly, the model was run again with the 218 georeferenced households from the five villages which included 727 individuals. The linear multiple regression analysis (R^2: 0.767, AIC: 566.7) showed that only BSI (1.3208) and Ind_Numb (0.7361) remained significant, while MSI and ORCDRC were no longer significant. The following variables also significantly contributed to the model ($p<0.05$): temperature (0.5163), pH index measured in water solution (Phihox) (-0.8366), BLDFIE (0.0090) and NDVI (4.1564). BSI, Ind_Numb, temperature, BLDFIE and NDVI showed a positive association with the risk of infection, while Phihox showed a negative association.

In other words, if the land surrounding the household had denser and healthier surrounding vegetation, higher temperature, a greater surface of bare soil or fallow lands, and more compacted soils (higher bulk density), the probability of infection with hookworm increased. At the same time, the number of individuals present in a household was a determinant for the risk of hookworm infection, with the risk increasing as the number of individuals per household increased. On the contrary, the risk increased as the pH in the soil decreased, which means a more acidic than alkaloid pH would be favourable for the development of hookworm larvae in the soil.

Once the risk model for hookworm infected individuals per household was applied to the household taking into consideration the characteristics considered, the estimated number of infections per household was predicted and is presented in Figure 7A. There was a coincidence in the prediction with respect to the observed values (Fig. 7B). The few households that presented a different estimation than the one observed, overestimated the number of infected individuals and in no cases did it underestimate the risk of infection.

Discussion

The dominant STH found in all the villages was hookworm, except for Zenzelema where the dominant species was *S. stercoralis*, both of which are transmitted percutaneously. The other two STH species, *A. lumbricoides* and *T. trichiura*, which are transmitted by the faecal-oral route, were found at very low prevalences (6). The village with the highest prevalence of hookworm, Kurbi, also had the highest average number of individuals per household infected and the neighbouring village of Gedro was the only village with high intensity hookworm infection. In this study, the concept that around 20% of individuals harbour the higher intensity infections was confirmed (25(42)), since around 80% of the infections found were of light intensity.

Both Kurbi and Gedro were the villages with the higher number of households with infected individuals with respect to the other three villages. Both of these villages were located closer to Tana Lake and have no access roads, therefore they are more remote than the rest of the villages included in the study. An area in Gedro with higher values than expected was identified, for both the number of infected individuals and for the intensity of infection; this might be due to the accumulation of infections over time and might be related to the way the health system is set up in the area. Normally, health extension workers (HEW) from the health centre in Zenzelema are in charge of preventive health activities in the villages, house to house visits and also controls that take place in the health centre, mainly for women of reproductive age. The HEW are also in charge of MDA for PSAC but not for SAC since that program is implemented at the schools (8, 9). Therefore, access to the villages is important, with Zenzelema, Mazoria and Sesaberet having an easy access to the main road and to Bahir Dar City, while the situation of Kurbi and Gedro is challenging, especially in the rainy season, when lands are flooded and small rivers make access more difficult. In these scenarios, HEW are not able access these villages and the inhabitants themselves usually do not travel to the health centre in Zenzelema or to the hospital in Bahir Dar unless they are very sick. In this area, MDA with albendazole is scheduled every 6 months, in May and November (11). However, data on the coverage of the program is poor and punctual situations alter the schedule frequently (i.e. due to the SARS-CoV-2 pandemic, MDA was implemented in December 2020 in a house by house manner).

Gedro and Sesaberet were the villages with the greatest vegetation cover, soil bulk density and less hydric stress and construction index. On the contrary, Zenzelema had fewer infected individuals per household and lower intensity infections. The houses in this village presented lower vegetation cover, lower proportion of bare soil and lower soil bulk density, although they presented higher hydric stress and construction index. This is in agreement with what was observed in the field since Zenzelema is located near the main road that connects this area with the North of the country and it is the most urban of all the five villages included, with the houses adjacent to each other and with less vegetation cover.

Individuals which practiced open defecation and those that owned their land for farming had a significant higher number of hookworm eggs in their stool; this relationship between hookworm infection and farming has been previously described (26-29(43-46)). Additionally, in the current study, those individuals that did not have electricity as well as those that used water from treated boreholes for cooking and handwashing also had a significant number of hookworm eggs in there stool. Unsafe water has also been previously associated with STH infection (18, 19, 30(47)). As part of the prevention activities performed by HEW in the different villages included in this study, are those related to the promotion of treatment of boreholes with chlorine. The finding that treated boreholes are associated with greater hookworm infection should be noted in order to revise the treatment of boreholes with respect to correct dosing of chlorine, safe storage/handling of water at the household level and the existence of a fence or cover to protect the borehole and avoid recontamination. The placement of the latrine and borehole should also be taken into consideration to avoid contamination of the water with faecal material.

The relationship between STH and a lack of sanitation and hygiene has been previously reported in Ethiopia with both positive (19, 23, 26(43), 30-32(47-49)) and negative associations (33(50)). Moreover, the relationship between a lack of electricity and the presence of intestinal parasite infection has also been found in other studies (20, 34(51)). In this study adults showed statistically higher hookworm egg counts than children; this confirms that for hookworm, adults tend to have higher intensities of infection contrary to what is observed for *A. lumbricoides* and *T. trichiura* (35(52)). Moreover, adults are not usually targeted for STH MDA so they may accumulate the infection over time and continue contaminating the

environment. Although the intensity of infection was influenced by the sanitary conditions of the household, the probability of infection with hookworm did not seem to be determined by socioeconomic conditions (22), except for the number of individuals per household, since infection tends to be more prevalent when there's overcrowding (18, 21, 36(53), 37(54)).

Nonetheless, infection does seem to be related to the environmental conditions around the household. The infection was more probable in households surrounded by a combination of vigorous vegetation and bare soil, high temperatures and compacted soils (high bulk density) with more acidic pH (15, 17, 38-40(25, 55-56)). This coincides with the findings of this study, since Gedro was the village that presented the highest number of infected individuals and higher intensity infections and was located in an area with greater spatial vegetation cover, compacted soil and lower hydric stress and construction index. This has already been observed in other studies with respect to the precipitation, temperature and vegetation cover (13, 41(57), 42(58)) but also with respect to the pH since hookworms can tolerate a pH range of 4.6 to 9.4 were they are still able to hatch and infect (43(59)). The presence of vigorous vegetation also favours the act of open defecation since it gives the individual privacy.

A coincidence in the prediction is observed, with a lack of an underestimation of the risk, even though a few households manifest an overestimation of the number of individuals infected. Although the value of precision of the model is acceptable, it would be interesting to analyse how the incorporation of the predictive variables of different factors and different spatial resolutions modify the performance of the prediction. The model generated would allow predicting the numbers of infected individuals per household in the study area as more predictive variables are added and updated. The estimated variables obtained from satellites are easily updatable in a frequent manner, while the variables obtained in the field (i.e. number of infected individuals per household) are relatively static. Therefore, a probability infection map that is frequently updated may be obtained so as to guide the health authorities in order to be able to plan actions for the prevention and control of hookworm and other soil-transmitted helminths.

Certain limitations of the study might have influenced the results, since not all nine villages from the area were included in the study and not all houses participated in the extended household questionnaire.

The use of environmental variables with high resolution, with a 30 meter radius around the household, together with the infection and intensity data evidenced that for the establishment of hookworm infection a suitable environment is necessary. Moreover, socioeconomic and behavioural parameters (such as source of water or practice of open defecation) seem to influence the intensity of the infection once it has already been established.

Acknowledgments

We would like to thank the Amhara National Regional State Health Bureau in Bahar Dar for its collaboration and support in this study. We appreciate the support of the Zenzelema Health Center director Mr. Tadesse Meseret Kokeb and all the staff for their collaboration. We are most grateful to the community leaders for facilitating the participation and contact with the community. We would also like to thank Marcelo Abril and Marcelo Scavuzzo for their institutional support, directors of Fundación Mundo Sano and Instituto Gulich, respectively.

Consent for publication

Not applicable.

Availability of data and materials

All data generated or analysed during this study are included in this published article [and its supplementary information files].

Competing interests

The authors declare that they have no competing interests.

Funding

This study was funded by Fundación Mundo Sano and Instituto de Salud Carlos III. The funders had no roles in the design of the study or collection, analysis and interpretation of the data. CMS has a PhD scholarship from Consejo Nacional de Investigaciones Científicas y Técnicas (CONICET).

Author´s contributions

M.A., A.A.A., Z.H., A.B. and M.V.P. designed the research, M.A. and A.A.A. organized and performed all the field research, S.L. collected all the remote sensing data and images and elaborated the figures, S.L., C.M.S. and M.V.P. analysed data and performed the statistical analysis, and M.A., S.L. and M.V.P. wrote the paper while A.A.A., Z.H. and M.V.P. edited it. All authors read and approved the final manuscript.

Competing interests

The authors declare no competing interests.

Data availability

All data generated or analysed during this study are included in this published article.

References

1. Hotez PJ, Kamath A. Neglected tropical diseases in Sub-Saharan Africa: review of their prevalence, distribution, and disease burden. PLoS Negl Trop Dis. 2009;3(8): e412. doi:10.1371/journal.pntd.0000412.

2. Mengitsu B, Shafi O, Kebede B, Kebede F, Worku DT, Herero M, et al. et al. Ethiopia and its steps to mobilize resources to achieve 2020 elimination and control goals for neglected tropical diseases: Spider webs joined can tie a lion. Int Health. 2016;8(Suppl 1): i34–i52. doi:10.1093/inthealth/ihw007.

3. Deribe K, Meribo K, Gebre T, Hailu A, Ali A, Aseffa A, et al. The burden of neglected tropical diseases in Ethiopia, and opportunities for integrated control and elimination. Parasit Vectors. 2012;5: 240. doi:10.1186/1756-3305-5-240.

4. Abera B, Alem G, Yimer M, Herrador Z. Epidemiology of soil-transmitted helminths, *Schistosoma mansoni,* and haematocrit values among schoolchildren in Ethiopia. J Infect Dev Ctries. 2013;7(3): 253-260. doi:10.3855/jidc.2539.

5. Amor A, Rodriguez E, Saugar JM, Arroyo A, López-Quintana B, Abera B, et al. High prevalence of *Strongyloides stercoralis* in school-aged children in a rural highland of north-western Ethiopia: the

role of intensive diagnostic work-up. Parasit Vectors. 2016;9: 617. doi:10.1186/s13071-016-1912-8.

6. Aramendia AA, Anegagrie M, Zewdie D, Dacal E, Saugar JM, Herrador Z, et al. Epidemiology of intestinal helminthiases in a rural community of Ethiopia: Is it time to expand control programs to include *Strongyloides stercoralis* and the entire community? PLoS Negl Trop Dis. 2020;14(6): e0008315. doi:10.1371/journal.pntd.0008315.

7. Terefe Y., Ross K., Whiley H. Strongyloidiasis in Ethiopia: systematic review on risk factors, diagnosis, prevalence and clinical outcomes. Infect Dis Poverty. 2019;8(1): 53. doi:10.1186/s40249-019-0555-3.

8. FMOH (Federal Democratic Republic of Ethiopia Ministry of Health). Ethiopia national master plan for neglected tropical diseases (2013–2015). Addis Ababa, Ethiopia. (Federal Democratic Republic of Ethiopia Ministry of Health, 2013). http://www.ntdenvision.org/sites/default/files/docs/national_ntd_master_plan_ethiopia_2013-2015_1.pdf. Accessed 30 June 2020.

9. Federal Democratic Republic of Ethiopia Ministry of Health, Schistosomiasis and soil-transmitted helminths national action plan. Addis Ababa, Ethiopia (Federal Democratic Republic of Ethiopia Ministry of Health, 2014).

10. Negussu N, Mengistu B, Kebede B, Deribe K, Ejigu E, Tadesse G, et al. Ethiopia schistosomiasis and soil-transmitted helminthes control programme: progress and prospects. Ethiop Med J. 2017;55(Suppl 1): 75–80.

11. Federal Democratic Republic of Ethiopia Ministry of Health, Ethiopia national master plan for neglected tropical diseases (2016-2020). Second Edition. (Federal Democratic Republic of Ethiopia Ministry of Health, Addis Ababa, Ethiopia, 2016).

12. Brooker S., Michael E. The potential of geographical information systems and remote sensing in the epidemiology and control of human helminth infections. Adv Parasitol. 2004;47: 245-288. doi:10.1016/s0065-308x(00)47011-9.

13. Alvarez Di Fino E, Rubio J, Abril M, Porcasi X., Periago MV. Risk map development for soil-transmitted helminth infections in Argentina. PLOS Negl Tropical Dis. 2020;14(2): e0008000. doi:10.1371/journal.pntd.0008000.

14. Chammartin F, Scholte RG, Malone JB, Bavia ME, Nieto P, Utzinger J, et al. Modelling the geographical distribution of soil-transmitted helminth infections in Bolivia. Parasit Vectors. 2013;6: 152. doi:10.1186/1756-3305-6-152.

15. Chaiyos J, Suwannatrai K, Thinkhamrop K, Pratumchart K, Sereewong C, Tesana S, et al. MaxEnt modeling of soil-transmitted helminth infection distributions in Thailand. Parasitol Res. 2018;117(11): 3507–3517. doi:10.1007/s00436-018-6048-7.

16. Lai YS, Zhou XN, Utzinger J., Vounatsou P. Bayesian geostatistical modelling of soil-transmitted helminth survey data in the People's Republic of China. Parasit Vectors. 2013;6: 359. doi:10.1186/1756-3305-6-359.

17. Oluwole AS, Ekpo UF, Karagiannis-Voules DA, Abe EM, Olamiju FO, Isiyaku S, et al. Bayesian geostatistical model-based estimates of soil-transmitted helminth infection in Nigeria, including annual deworming requirements. PLoS Negl Trop Dis. 2015;9(4): e0003740. doi:10.1371/journal.pntd.0003740.

18. Chin YT, Lim YA, Chong CW, Teh CS, Yap IK, Lee SC, et al. Prevalence and risk factors of intestinal parasitism among two indigenous sub-ethnic groups in Peninsular Malaysia. Infect Dis Poverty. 2016;5: 77. doi: 10.1186/s40249-016-0168-z.

19. Molla E, Mamo, H. Soil-transmitted helminth infections, anemia and undernutrition among school children in Yirgacheffee, South Ethiopia. BMC Res Notes. 2018;11: 585. doi:10.1186/s13104-018-3679-9.

20. Periago MV, García R, Astudillo O, Cabrera M, Abril M. Prevalence of intestinal parasites and the absence of soil-transmitted helminths in Añatuya, Santiago del Estero, Argentina. Parasit Vectors. 2018;11(1): 638. doi:10.1186/s13071-018-3232-7.

21. Romero-Sandoval N, Ortiz-Rico C, Sánchez-Pérez HJ, Valdivieso D, Sandoval C, Pástor J, et al. Soil transmitted helminthiasis in indigenous groups. A community cross sectional study in the Amazonian southern border region of Ecuador. BMJ Open. 2017;7(3): e013626. doi:10.1136/bmjopen-2016-013626.

22. Abdia M, Nibretb E,Munshea A. Prevalence of intestinal helminthic infections and malnutrition among schoolchildren of the Zegie Peninsula, northwestern Ethiopia. J Infect Public Health. 2017;10: 84-92. doi:10.1016/j.jiph.2016.02.009.

23. Muluneh C, Hailu T, Alemu G. Prevalence and associated factors or soil-transmitted helminth infection among children living with and without open defecation practices in Northwest Ethiopia: A comparative cross-sectional study. Am. J. Trop. Med and Hyg. 2020;103(1): 266-272. doi:10.4269/ajtmh.19-0704.

24. Nute AW, Endeshaw T, Stewart AEP, Sata E, Bayissasse B, Zerihun M, et al. Prevalence of soil-transmitted helminths and *Schistosoma mansoni* among a population-based sample of school-age children in Amhara region, Ethiopia. Parasit Vectors. 2018;11(1): 431. doi:10.1186/s13071-018-3008-0.

25. Knopp S, Mohammed KA, Simba Khamis I, Mgeni AF, Stothard JR, Rollinson D, et al. Spatial distribution of soil transmitted helminths, including *Strongyloides stercoralis*, among children in Zanzibar. Geospat Health. 2008;3(1): 47–56. doi:10.4081/gh.2008.231.

26. Chammartin F, Scholte RG, Guimaraes LH, Tanner M, Utzinger J, Vounatsou P. Soil-transmitted helminth infection in South America: a systematic review and geostatistical meta-analysis. Lancet Infect Dis. 2013;13(6): 507–518. doi:10.1016/S1473-3099(13)70071-9.

27. Chammartin F, Guimaraes LH, Scholte RG, Bavia ME, Utzinger J, Vounatsou P. Spatio-temporal distribution of soil-transmitted helminth infections in Brazil. Parasit Vectors. 2014;7: 440. doi: 10.1186/1756-3305-7-440.

28. Khieu V, Schar F, Forrer A, Hattendorf J, Marti H, Duong S, et al. High prevalence and spatial distribution of *Strongyloides stercoralis* in rural Cambodia. PLoS Negl Trop Dis. 2014; 24921627. doi:10.1371/journal.pntd.0002854.

29. Owada K, Lau CL, Leonardo L, Clements ACA, Yakob L, Nielsen M, et al. Spatial distribution and populations at risk of *A. lumbricoides* and *T. trichiura* co-infections and infection intensity classes: an ecological study. Parasit Vectors. 2018;11(1): 535. doi:10.1186/s13071-018-3107-y.

30. Pullan RL, Bethony JM, Geiger SM, Cundill B, Correa-Oliveira R, Quinnell RJ, et al. Human helminth co-infection: analysis of spatial patterns and risk factors in a Brazilian community. PLoS Negl Trop Dis. 2008;2(12): e352. doi:10.1371/journal.pntd.0000352.

31. Soares Magalhaes RJ, Langa A, Pedro JM, Sousa-Figueiredo JC, Clements AC, Vaz Nery S. Role of malnutrition and parasite infections in the spatial variation in children's anaemia risk in northern Angola. Geospat Health. 2013;7(2): 341–354. doi:10.4081/gh.2013.91.

32. Rouse JW, Haas RH, Schell JA, Deering DW. *Monitoring vegetation systems in the Great Plains with ERTS*. In: NASA Goddard Space Flight Center 3d ERTS-1. https://ntrs.nasa.gov/search.jsp?R=19740022614 2020-07-27T22:27:18+00:00Z (1974).

33. Hunt Jr. ER, Rock BN. Detection of changes in leaf water content using near-and middle-infrared reflectances. Remote Sens Environ. 1989;30(1): 43-54. doi:10.1016/0034-4257(89)90046-1.

34. Welikhe P, Quansah JE, Fall S., McElhenney W. Estimation of soil moisture percentage using Landsat-based moisture stress index. J Remote Sens & GIS. 2017;6(2): 1000200. doi:10.4172/2469-4134.1000200.

35. Zha Y, Gao J., Ni S. Use of normalized difference built-up index in automatically mapping urban areas from TM imagery. Int J Remote Sens. 2013;24(3): 583-594. doi:10.1080/01431160304987.
36. Rikimaru A, Roy PS, Miyatake S. Tropical forest cover density mapping. Trop Ecol. 2002;43(1): 39-47.
37. ISRIC World Soil Information (2020). https://www.isric.org/projects/africa-soil-information-service-afsis. Accessed 25 April 25 2020.
38. Miller RO, Kissel DE. Comparison of soil pH methods on soils of North America. Soil Sci Soc Am J. 2010;74(1): 310-316.
39. Kulldorff, M. Information management services SaTScan: software for the spatial and space–time scan statistics, version 4.0 [computer program]. (2003). Available: http://www.satscan.org/. Accessed 30 June 2020.
40. Kulldorff M, Huang L, Konty K. A scan statistic for continuous data based on the normal probability model. Int J Health Geogr. 2009; doi:10.1186/1476-072X-8-58.
41. Montresor A, Crompton DWT, Hall A, Bundy DAP, Savioli L. Guidelines for the evaluation of soil-transmitted helminthiasis and schistosomiasis at community level: a guide for managers of control programmes. World Health Organization (1998). https://apps.who.int/iris/handle/10665/63821.
42. Bundy DAP. Population ecology of intestinal helminth infections in human communities. Philos Trans R Soc Lond B Biol Sci. 1998;321(1207): 405-420. doi:10.1098/rstb.1988.0100.
43. Kaliappan SP, George S, Francis MR, Kattula D, Sarkar R, Minz S, et al. Prevalence and clustering of soil-transmitted helminth infections in a tribal area in southern India. Trop Med Int Health. 2013; 18(12): 1452–1462. doi:10.1111/tmi.12205.
44. Loukouri A, Méité A, Kouadio OK, Djè NN, Trayé-Bi G, Koudou BG, et al. Prevalence, intensity of soil-transmitted helminths, and factors associated with infection: importance in control program

with ivermectin and albendazole in Eastern Côte d'Ivoire. J Trop Med. 2019; 7658594. doi:10.1155/2019/7658594.

45. Matthys B, Tschannen AB, Tian-Bi NT, Comoe H, Diabate S, Traore M, et al. Risk factors for *Schistosoma mansoni* and hookworm in urban farming communities in western Cote d'Ivoire. Trop Med Int Health. 2007;12(6): 709-723. doi:10.1111/j.1365-3156.2007.01841.x.

46. Shah HA, Huxley P, Elmes J, Murray KA. Agricultural land-uses consistently exacerbate infectious disease risks in Southeast Asia. Nat Commun. 2019;10(1): 4299. doi:10.1038/s41467-019-12333-z.

47. Tekalign E, Bajiro M, Ayana M, Tiruneh A, Belay T. Prevalence and intensity of soil-transmitted helminth infection among rural community of Southwest Ethiopia: a community-based study. Biomed Res Int. 2019;3687873. doi:10.1155/2019/3687873.

48. Anunobi JT, Okoye IC, Aguzie IO, Ndukwe YE, Okpasuo OJ. Risk of soil-transmitted helminthiasis among agrarian communities of Kogi State, Nigeria. Ann Glob Health. 2019;85(1): 120. doi:10.5334/aogh.2563.

49. Grimes JET, Tadesse G, Mekete K, Wuletaw Y, Gebretsadik A, French MD, et al. School water, sanitation, and hygiene, soil-transmitted helminths, and schistosomes: national mapping in Ethiopia. PLoS Negl Trop Dis. 2016;10(3): e0004515. doi:10.1371/journal.pntd.0004515.

50. Oswald WE, Stewart AEP, Kramer MR, Endeshaw T, Zerihun M, Melak B, et al. Association of community sanitation usage with soil-transmitted helminth infections among school-aged children in Amhara Region, Ethiopia. Parasit Vectors. 2017;10: 91. doi:10.1186/s13071-017-2020-0.

51. Morales-Espinoza EM, Sánchez-Pérez HJ, García-Gil MM, Vargas-Morales G, Méndez-Sánchez JD, Pérez-Ramirez M. Intestinal parasites in children, in highly deprived areas in the border region of Chiapas, Mexico. Salud Publica Mex. 2003;45(5): 379-388. doi:10.1590/s0036-36342003000500008.

52. Truscott JE, Hollingsworth TD, Brooker SJ, Anderson RM. Can chemotherapy alone eliminate the transmission of soil transmitted helminths? Parasit Vectors. 2014;7(1): 266. doi:10.1186/1756-3305-7-266.

53. Milano AM, Oscherov EB, Palladino AC, Bar AR. Children enteroparasitosis in north east Argentine urban area. Medicina (B Aires). 2007;67: 238–42 (in Spanish).

54. Parija SC, Chidambaram M, Mandal J. Epidemiology and clinical features of soil-transmitted helminths. Trop Parasitol. 2017;7(2): 81-85. doi:10.4103/tp.TP_27_17.

55. Mudenda NB, Malone JB, Kearney MT, Mischler PD, Nieto PM, McCarroll JC, et al. Modelling the ecological niche of hookworm in Brazil based on climate. Geospat Health. 2012;6(3): S111-S123.

56. Scholte RGC, Schur N, Bavia ME, Carvalho EM, Chammartin F, Utzinger J, et al. Spatial analysis and risk mapping of soil-transmitted helminth infection in Brazil, using Bayesian geostatical models. Geospat Health. 2013;8(1): 97-110. doi:10.4081/gh.2013.58.

57. Ovutor O, Imafidor H, Awi-waadu GDW. Assessment of physico-chemical parameters of soils in fallowing farmlands and pit toilet environments as it affects the abundance of geohelminthes in Emohua Local Government Area, Rivers State, Nigeria. Annu Res Rev. 2017;14(3): 1-10. doi:10.9734/ARRB/2017/31546.

58. Sedionoto B, Wasessombat S, Punsawad C, Anamnart W. Environmental factors and prevalence of hookworm infection and strongyloidiasis in Rural East Kalimantan, Indonesia. E3S Web of Conferences. 2019;125: 04001. doi:10.1051/e3sconf/201912504001.

59. World Health Organization. Integrated guide to sanitary parasitology. WHO Regional Office for the Eastern Mediterranean (2004).

Figure 1. Study area in Amhara Region, Ethiopia. The villages included in this study were Kurbi, Gedro, Mazoria, Sesaberet and Zenzelema, belonging to a rural district. The number of people (n) recruited per village as well as their age range in years (Y), average age (X) and distribution by sex (F = % female) are

noted in the box on the right hand corner. Map data © 2020 Openstreetmap, base map obtained through QuickMap Services QGIS plugin - QGIS Geographic Information System. Open Source Geospatial Foundation Project. http://qgis.osgeo.org.

Figure 2. An example of the villages included in the study with Mazoria (A) as an example of a more rural and isolated village and Zenzelema (B) as an example of a more urbanized village in the main road.

Figure 3. Spatial distribution of hookworm infection. A. Number of infected individuals per household. B. Mean EPG in each of the households for the five villages. GE: Gedro, KU: Kurbi, MA: Mazoria, SE: Sesaberet, ZE: Zenzelema. Map data © 2020 Google, base map obtained through QuickMap Services QGIS plugin - QGIS Geographic Information System. Open Source Geospatial Foundation Project. http://qgis.osgeo.org.

Figure 4. Spatial clusters of households with high value of mean EPG per household and number of infected individuals. The color of each household point refers to the mean EPG value. GE: Gedro, KU: Kurbi, MA: Mazoria, SE: Sesaberet, ZE: Zenzelema. Map data © 2020 Google, base map obtained through QuickMap Services QGIS plugin - QGIS Geographic Information System. Open Source Geospatial Foundation Project. http://qgis.osgeo.org.

Figure 5. Boxplots showing the association between the number of eggs per gram of feces (EPG) and characteristics of the households. A. Toilet Type. B. Presence/absence of electricity. C. Water source for cooking. D. Water source for handwashing. E. Age (Child refers to all those under 14 and Adult refers to all those 15 or older). F. Land property. MWU: Mann-Whitney U, KWH: Kruskal-Wallis H, p: significance test ($p<0.05$).

Figure 6. Spatial distribution of the different soil and environmental indexes analyzed. NDVI: Normalized Difference Vegetation Index. MSI: Moisture Stress Index. BSI: Bare Soil Index. NDBI: Normalized Difference Built-up Index. BLDFIE: Soil Bulk density. Map data © 2020 Google, base map obtained through

QuickMap Services QGIS plugin - QGIS Geographic Information System. Open Source Geospatial Foundation Project. http://qgis.osgeo.org.

Figure 7. Map of the number of the hookworm infected individuals predicted (A) and observed (B) per household in the five villages. GE: Gedro, KU: Kurbi, MA: Mazoria, SE: Sesaberet, ZE: Zenzelema. Map data © 2020 Google, base map obtained through QuickMap Services QGIS plugin - QGIS Geographic Information System. Open Source Geospatial Foundation Project. http://qgis.osgeo.org.

Figure 1

Click here to access/download;Figure;Figure 1.jpg

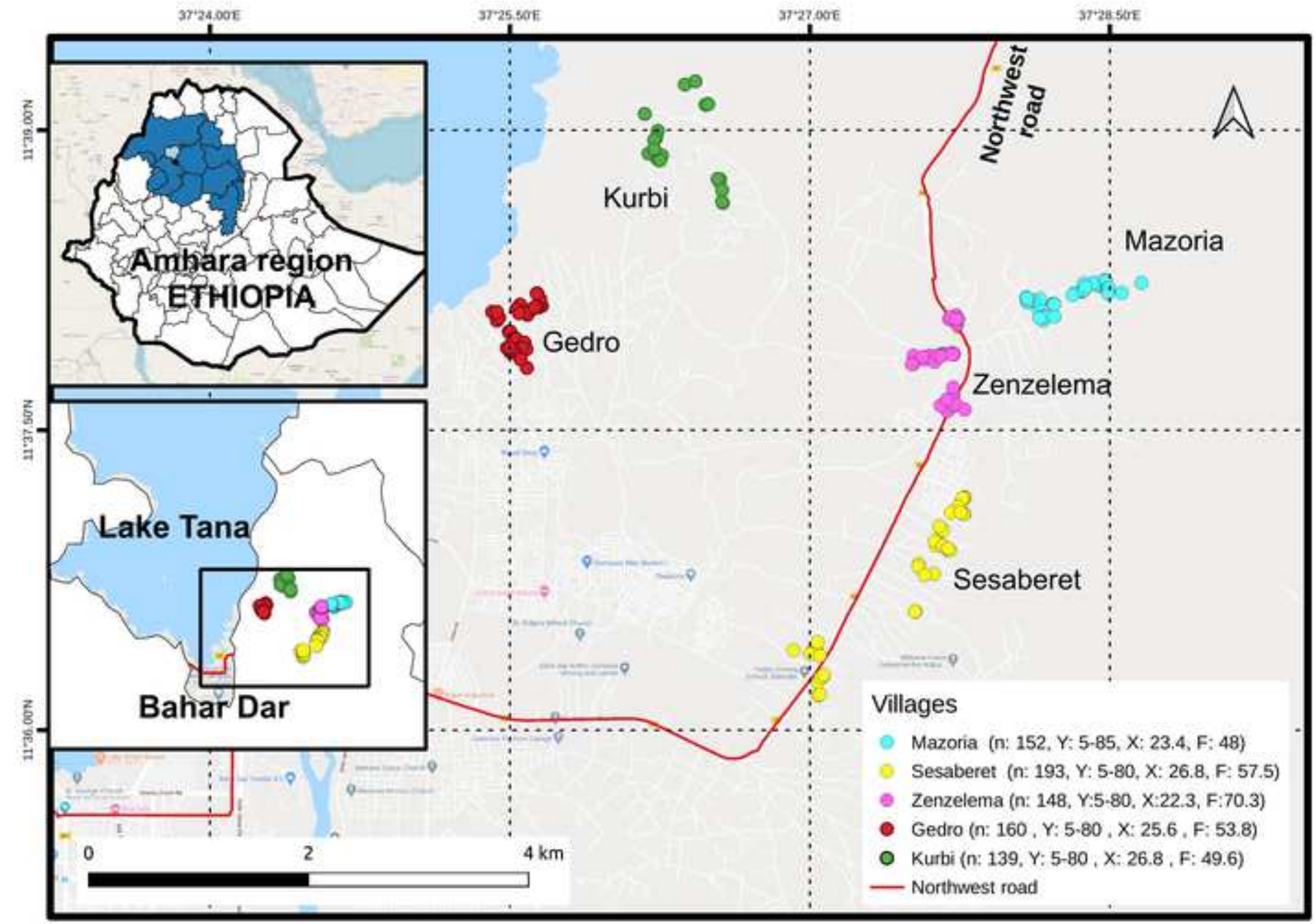

Figure 2

Click here to access/download;Figure;Figure 2.jpg

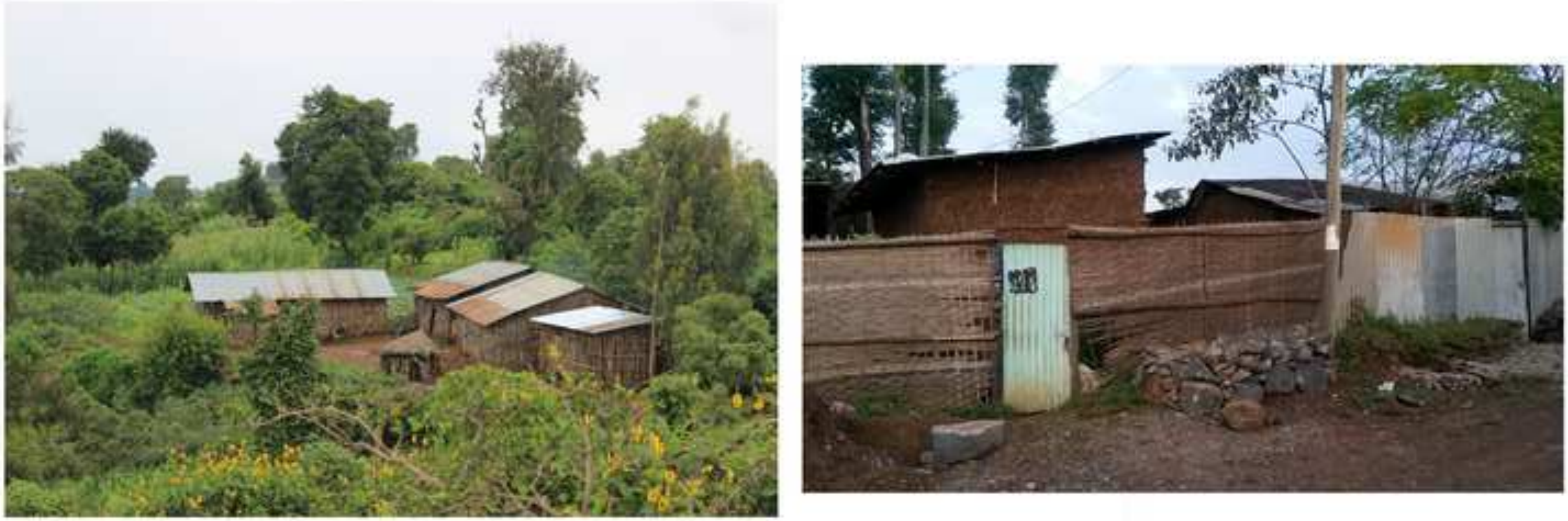

A B

Figure 3

Click here to access/download;Figure;Figure 3.jpg

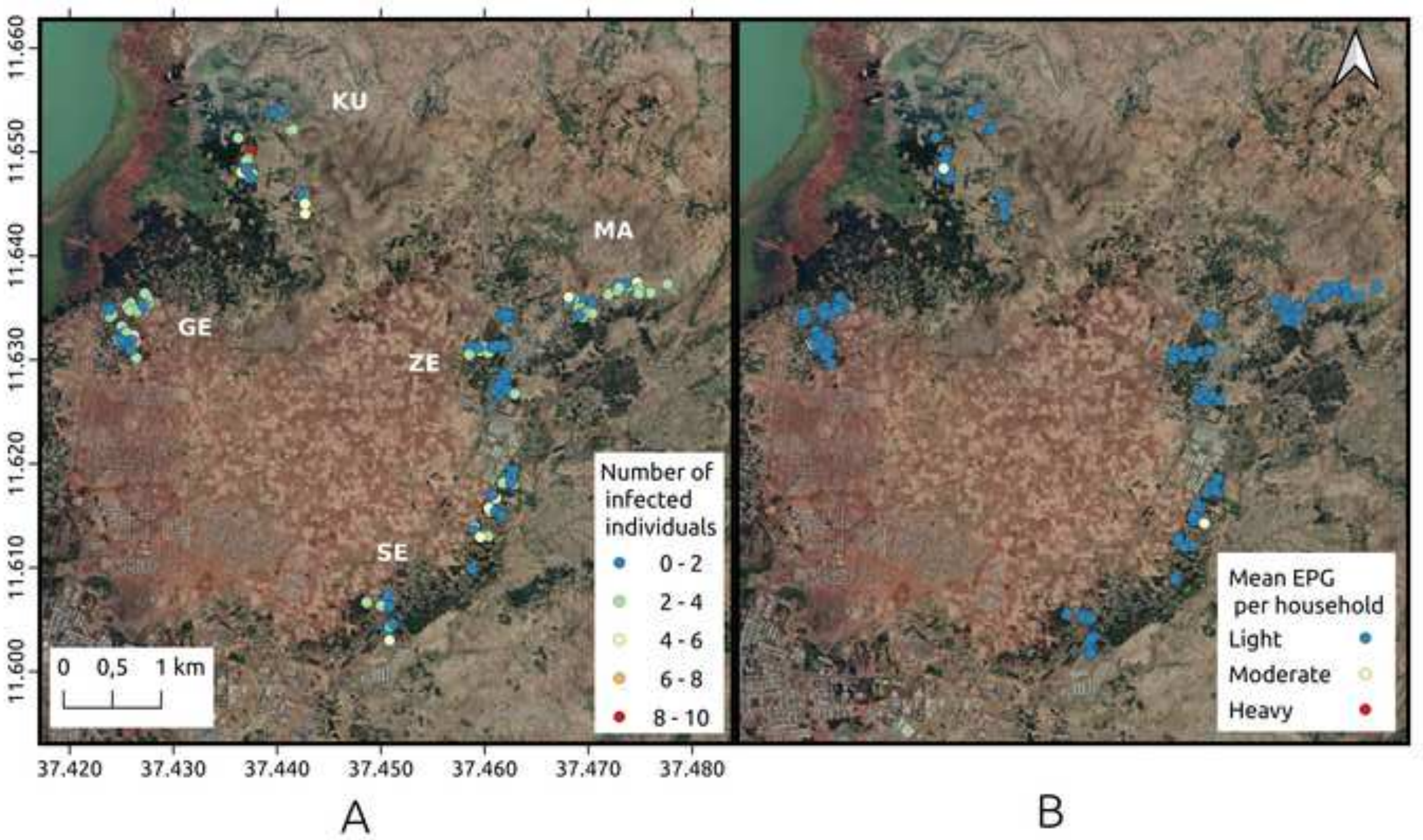

Figure 4

Click here to access/download;Figure;Figure 4.jpg

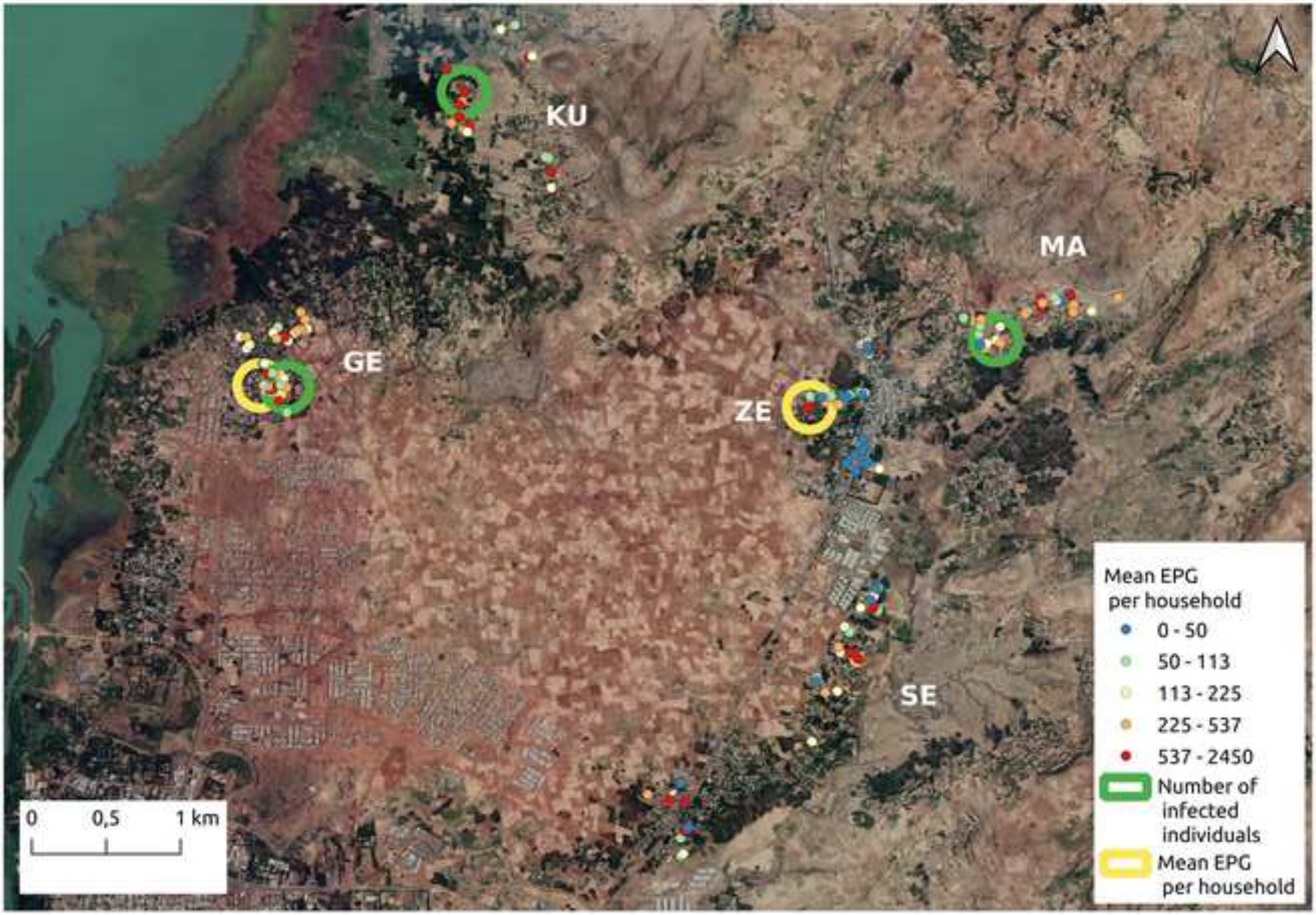

Figure 5

Click here to access/download;Figure;Figure 5.jpg

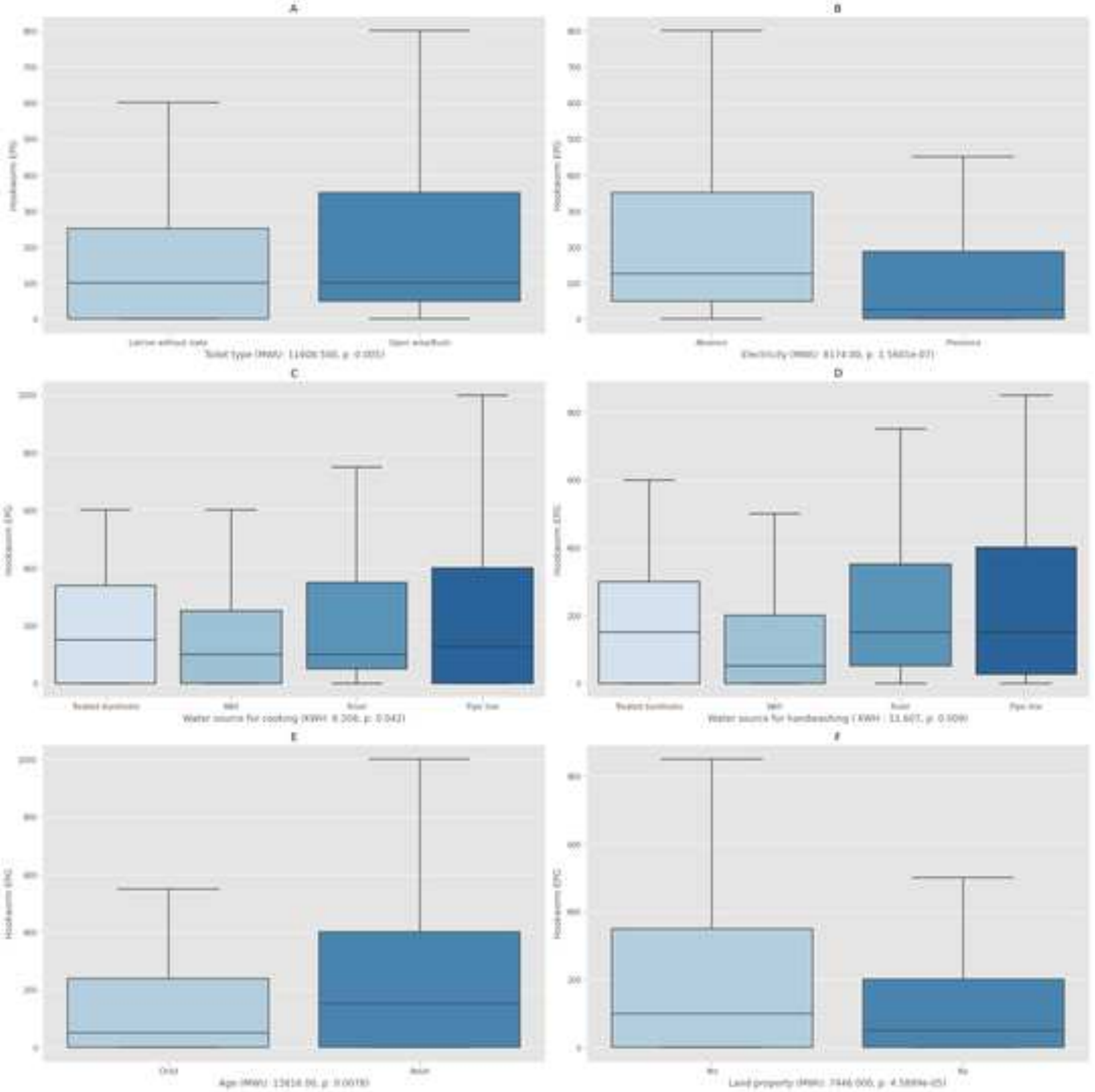

Figure 6

Click here to access/download;Figure;Figure 6.jpg

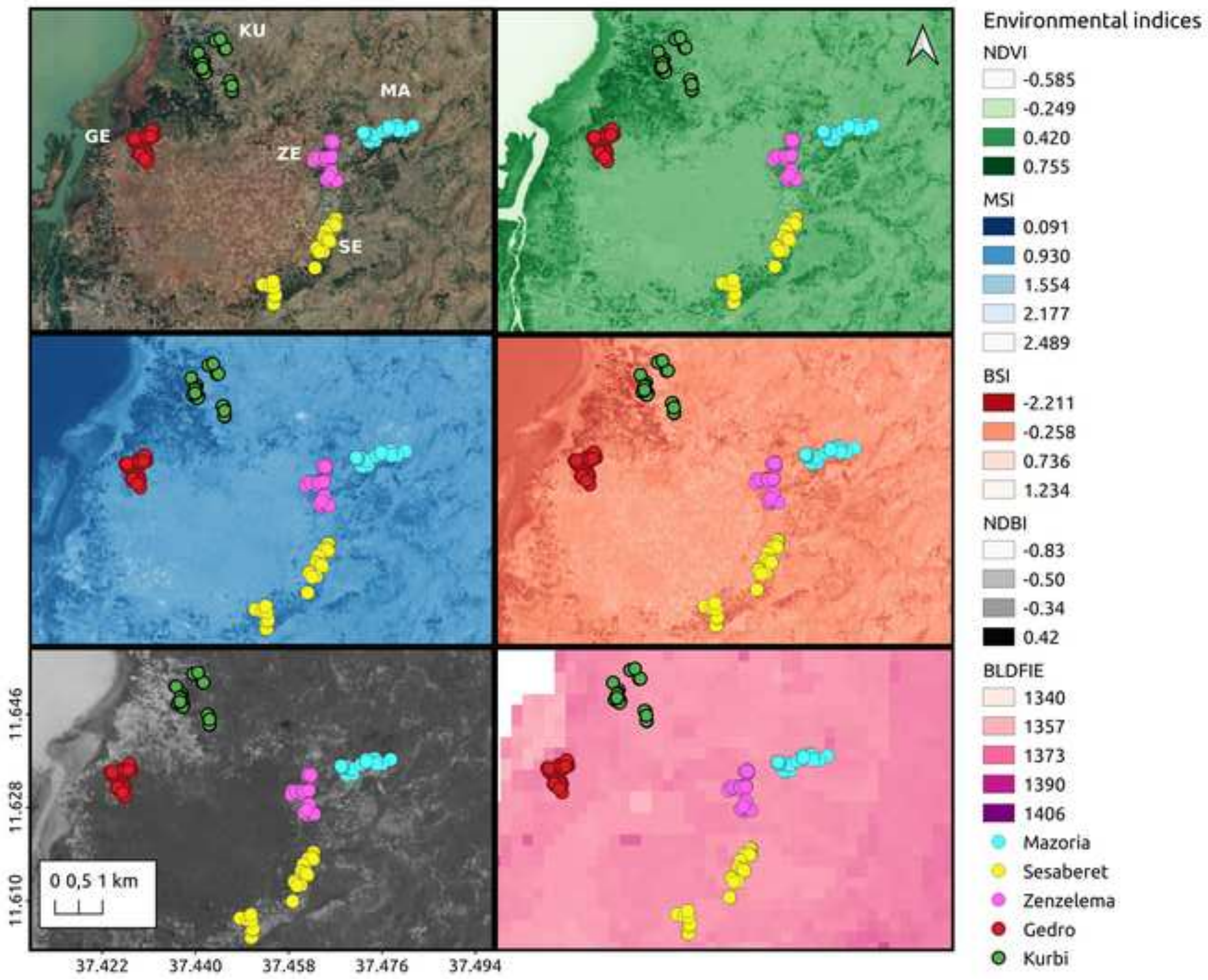

Figure 7

Click here to access/download;Figure;Figure 7.jpg

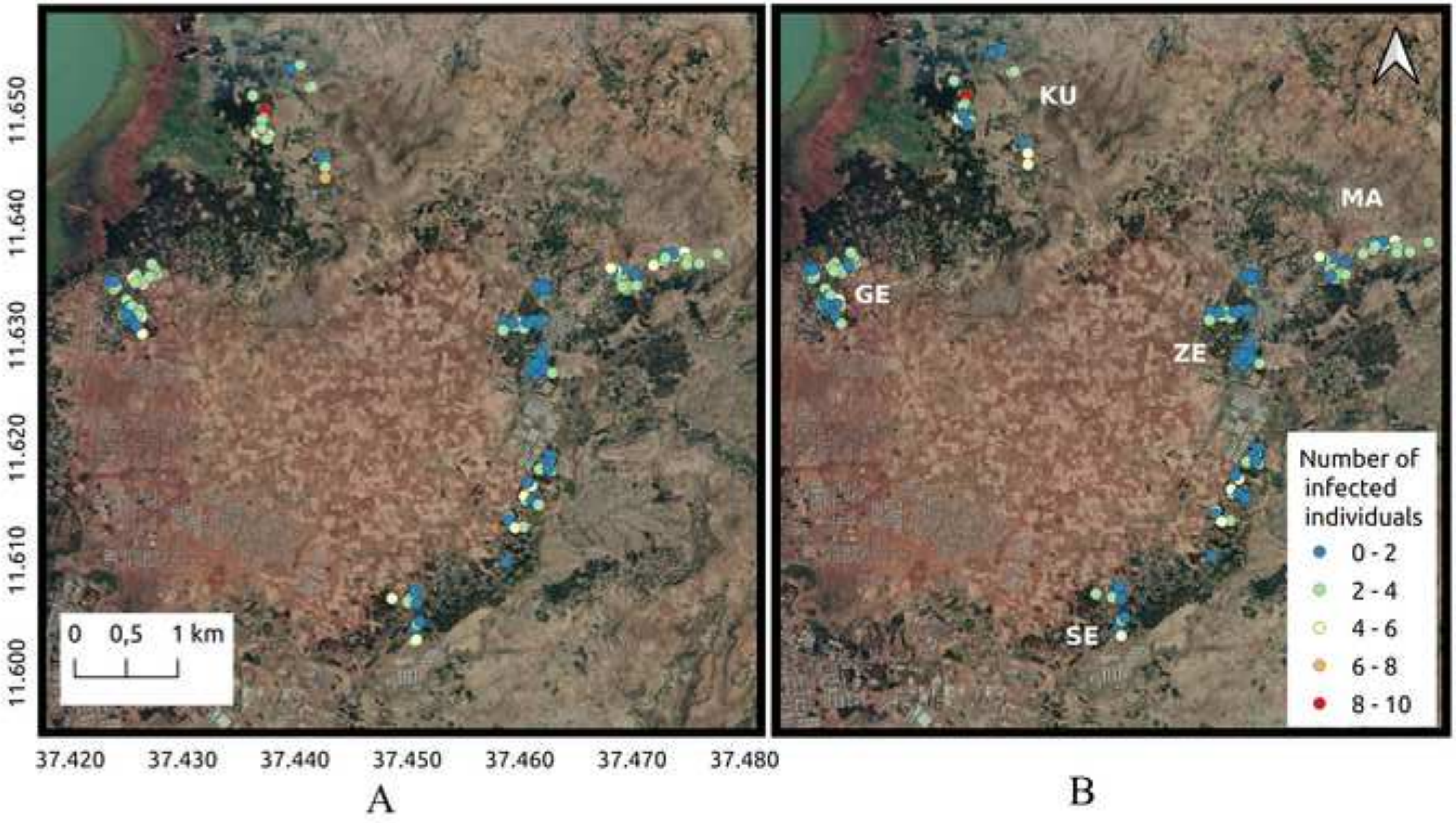

Supplementary Table

Click here to access/download
Supporting Information
Supplementary Table 1.docx
