# Supplementary material for: Environmental characteristics around the household and their association with hookworm infection in rural communities from Bahir Dar, Amhara Region, Ethiopia
Source: PLoS Negl Trop Dis. 2021 Jun 22;15(6):e0009466. doi: 10.1371/journal.pntd.0009466 (PMC8219153; doi:10.1371/journal.pntd.0009466)
Supplement: Attachment — Submitted filename: H Dolo review on the manuscript09.03.21.docx [file pntd.0009466.s002.docx]

General comment on the manuscript

1. Summary of the research

Despite the ongoing mass drug administration there is bottle neck about the elimination STH in several countries and this type of assessment done by María Victoria Periago et al. is useful to explore the environmental characteristics and their implications on NTD.

This is very interesting areas of research on Neglected tropical diseases to study the impact of environment on the occurrence or presence of STH under ongoing mass drug administration with the goal to eliminate them in the near future. They came with very important findings showing that open defecation and lack of electricity, being adults are associated with hookworm infection. These findings add new knowledge in the field of NTDs research and will lead to reassess new strategies or improve existing strategies.

However, the authors should report also the few cases of STH before focusing on the hookworm found during their study.

My overall recommendation is structured as follows :

2. Major issues

The authors need to end up the discussion with very concise conclusion about their works and its implication for public health

3. Minor issues

Line 39 : more acidic pH this is vague please I will suggest to give a range or more precision

Author summary

Line 47 : I will suggest to precisely define the environment element that the authors assessed in their work

Line 52-53: please revise to keep the

“intensity of hookworm infection was associated to certain socioeconomic conditions such as the practice of open defecation and a lack of electricity”

Key words:

Line 57: I will suggest removing Soil-transmitted helminths

Introduction

Line 61-62 and line 65: please add the abbreviation at first appearance Soil-Transmitted

Helminthiases

Methods

Please add subheading to define or describe the terms like woredas???, and kebeles???

Data collection

Line 127: “using WHO standardized surveys” I think this needs reference

Line 129: ….. while the head of the family was also asked to answer a household questionnaire. I think it will be good to explain slightly this household question for the heads

Line 133: Stool samples were processed as previously described (6) to determine presence and intensity of hookworm infection. Actually, in ref 6 they used 3 three methods. IF you used also the 3 good to reported before referring to 6.

Results

Line 206 -208: “Stool samples were processed as previously described (6) to determine presence and intensity of hookworm infection. “ I will suggest to remove this from the results section to avoid any confusion.

Line 213-214: Table 4. Infection status of the participants of the study from the five villages, Kurbi, Gedro, Mazoria, Sesaberet and Zenzelema (Amhara State, Ethiopia). I will suggest the following “Hookworm infection status in study participants from the five villages, Kurbi, Gedro, Mazoria, Sesaberet and Zenzelema, Amhara State, Ethiopia

Table 4 needs also a formatting e.g presenting this in landscape

Line 231-233: I will suggest removing this from the results section, will fit better to discussion

Line 272: needs space after (BLDFIE)

Discussion

Line 342: lack of electricity associated with STH infection need cautious interpretation because this can stand as confounders

Also during the discussion I will suggest the authors to discussion the implication of their findings in the currents strategies against STH or Hookworm
