# Supplementary material for: Environmental characteristics around the household and their association with hookworm infection in rural communities from Bahir Dar, Amhara Region, Ethiopia
Source: PLoS Negl Trop Dis. 2021 Jun 22;15(6):e0009466. doi: 10.1371/journal.pntd.0009466 (PMC8219153; doi:10.1371/journal.pntd.0009466)
Supplement: Attachment — Submitted filename: Review of the manuscripts response to reviewers.docx [file pntd.0009466.s004.docx]

Reviewer's Responses to Questions

First of all, we would like to thank the reviewers for they thorough analysis, comments and suggestions. We have corrected the errors detected and your input has greatly improved the flow and comprehension of the study presented.

General comment on the manuscript

Reviewer #1:

1. Summary of the research

Despite the ongoing mass drug administration there is bottle neck about the elimination STH in several countries and this type of assessment done by María Victoria Periago et al. is useful to explore the environmental characteristics and their implications on NTD.

This is very interesting areas of research on Neglected tropical diseases to study the impact of environment on the occurrence or presence of STH under ongoing mass drug administration with the goal to eliminate them in the near future. They came with very important findings showing that open defecation and lack of electricity, being adults are associated with hookworm infection. These findings add new knowledge in the field of NTDs research and will lead to reassess new strategies or improve existing strategies.

However, the authors should report also the few cases of STH before focusing on the hookworm found during their study.

My overall recommendation is structured as follows:

2. Major issues

The authors need to end up the discussion with very concise conclusion about their works and its implication for public health

3. Minor issues

Line 39: more acidic pH this is vague please I will suggest to give a range or more precision

This was modified accordingly and a pH of 6.0 was specified.

Author summary

Line 47: I will suggest to precisely define the environment element that the authors assessed in their work

Thank you for your suggestion. The lines states “we aimed to explore the association between the environment, soil and socioeconomic characteristics most associated with the presence of hookworm infection.” Since this is the author summary, there is a limit of words that can be included and it should be made understandable to someone that does not work in the area, that is why we decided to leave it as a general statement, not only for the environmental characteristics but also for the soil and socioeconomic ones as well. Mainly due to the word count constraint.

Line 52-53: please revise to keep the

“intensity of hookworm infection was associated to certain socioeconomic conditions such as the practice of open defecation and a lack of electricity”

This was revised accordingly.

Key words:

Line 57: I will suggest removing Soil-transmitted helminths

This was removed as suggested.

Introduction

Line 61-62 and line 65: please add the abbreviation at first appearance Soil-Transmitted

Helminthiases

Since this is referring to the disease, Soil-Transmitted Helminthiasis and the first time we mention Soil-Transmitted Helminths is in line 65, we added the abbreviation in that line and not in line 61-62.

Methods

Reviewer #1:

Please add subheading to define or describe the terms like woredas???, and kebeles???

In the description of the study area we state “Ethiopia is a Federal Democratic Republic comprised of ten regional states and two administrative cities which are then subdivided into 95 zones and 839 administrative *woredas* or districts. *Woredas* are further divided into 16,253 *kebeles*, the smallest administrative unit.” This is described in lines 101-104.

Data collection

Line 127: “using WHO standardized surveys” I think this needs reference

A reference was added as suggested.

Line 129: ….. while the head of the family was also asked to answer a household questionnaire. I think it will be good to explain slightly this household question for the heads.

In lines 152 it states “Several socioeconomic variables were measured related to the number of individuals per household, income, toilet type, water source, presence of electricity, etc. These are further detailed in the result section.” All the variables measured are also listed in Supplementary Table 1.

Line 133: Stool samples were processed as previously described (6) to determine presence and intensity of hookworm infection. Actually, in ref 6 they used 3 three methods. If you used also the 3 good to reported before referring to 6.

This was modified to reflect that the infection data used was the one obtained in reference 6 (now 8), therefore the methodology is exactly the one described in reference 6 (now 8), with all three methods.

Reviewer #2: It is necessary to inform how the parasitological examinations were performed. Was only the McMaster technique performed? It is necessary to describe in detail the laboratory procedures used for the diagnosis of hookworm infections.

This was modified to state more clearly that the infection prevalence and intensity used for the spatial analysis was the one published previously in reference 6 (now 8). Also, the three techniques used in that study were added to the current manuscript to specify precisely which techniques were used, so as to avoid confusion. The description of the laboratory techniques is specified with detail in reference 6 (now 8).

One of the dependent variables used in the analyzes was the "mean number of EPG per household". The use of means requires information on standard deviation. It also requires that the distribution be normal. Justify the use and adequacy of the "mean number of EPG per household" as a parasitological parameter.

Given your comment and that of reviewer #3, we have changed the use of the arithmetic mean of EPG per household to the geometric mean of EPG per household, this value does not follow a normal distribution and a 95% Confidence Interval was used instead.

Reviewer #3: The method section provides no details of the laboratory analysis. How were specimens collected? How many specimens were collected? How long and in what way were they stored and processed prior to analysis? Where was analysis performed? What methodology was used (MacMaster flotation is mentioned in the footnotes of table 1, but no details of how this was performed have been provided)? What was the level of competence of the microscopists and was any quality control performed such as re-analysis of a percentage of samples by a separate technician? This is a major flaw and must be addressed thoroughly.

This was modified to state more clearly that the infection prevalence and intensity used for the spatial analysis was the one published previously in reference 6 (now 8). Also, the three techniques used in that study were added to the current manuscript to specify precisely which techniques were used, so as to avoid confusion. The description of the laboratory techniques is specified with detail in reference 6 (now 8), with all the other information on processing and quality control.

--------------------

Results

Reviewer #1:

Line 206 -208: “Stool samples were processed as previously described (6) to determine presence and intensity of hookworm infection”. I will suggest to remove this from the results section to avoid any confusion.

This was removed as suggested.

Line 213-214: Table 4. Infection status of the participants of the study from the five villages, Kurbi, Gedro, Mazoria, Sesaberet and Zenzelema (Amhara State, Ethiopia). I will suggest the following “Hookworm infection status in study participants from the five villages, Kurbi, Gedro, Mazoria, Sesaberet and Zenzelema, Amhara State, Ethiopia.

This was modified as suggested.

Table 4 needs also a formatting e.g presenting this in landscape.

Noted.

Line 231-233: I will suggest removing this from the results section, will fit better to discussion.

This was moved to the discussion as suggested.

Line 272: needs space after (BLDFIE)

Noted.

Reviewer #2: In Table 4, in the line referring to No. of infected individuals by intensity as per MM [No.] , the sum of numbers in the intensity categories are sometimes not is equal to the total number of positives.

In Table 4, in addition to presenting "Mean number of infected individuals per household (mean)", it would be important to present the proportion of positive subjects among the total tested, per household.

These comments have been taken into consideration and the analysis of the data was re-processed and inconsistencies were revised.

Reviewer #3: The numbers in table 1 do not add up correctly and should be checked. Also, please use geometric mean instead of mean for average egg intensity counts.

The geometric mean was used throughout therefore Table 4 was modified and also checked for consistency. The use of the geometric mean also impacted Figures 3 and 4 so they were re-elaborated using the geometric mean instead of the arithmetic mean.

--------------------

Discussion

Reviewer #1:

Line 342: lack of electricity associated with STH infection need cautious interpretation because this can stand as confounders

Noted.

Also during the discussion, I will suggest the authors to discussion the implication of their findings in the currents strategies against STH or Hookworm.

The implications of the findings in the currents strategies for MDA have been added throughout the discussion.

Conclusions

Reviewer #1: the manuscript needs a conclusion section which is missing in this current version

A conclusion has been added.

Reviewer #2: The correlation between farming and higher hookworm infection intensity should be better discussed. The permanence of hookworm infections despite the significant reduction in the prevalence of infections by Ascaris and Trichuris has been observed in several regions, in other countries, with the same characteristics. This finding, that is, the different epidemiological behavior of percutaneously and orally transmitted STHs needs to be better addressed in the discussion.

Thank you for your comment, a statement was added in the discussion to address this matter (lines 364-366).

It is necessary to discuss the determinants of the practice of open defecation. Since this practice is closely related to the frequency and intensity of hookworm infections, it is necessary to understand it. Are there cultural determinants? Do people who practice subsistence farming need to defecate in the workplace? What alternatives to open defecation can be proposed to populations living in this socio-demographic and environmental scenario?

These are very important questions and a statement was added in the discussion together with references on the matter. Lines 396 to 399.

Reviewer #3: There is no final conclusion paragraph and this should be added.

A conclusion has been added.

The statistically significant associations of environmental factors with STH infection (e.g. borehole treatment) have been accepted as causative, but how they might be causative is not well addressed, could some of these associations be related to other factors which are actually causing the high rates of hookworm infection?

We present statistically significant associations of environmental factors with hookworm infection and also present significant associations between the intensity of hookworm infection and certain socioeconomic factors such as the use of borehole water for drinking. This by no means are accepted as causal. As explained in the discussion, the boreholes are usually treated by the Health Extension Workers, therefore, given people remain infected, the water is probably either not being treated, or not being properly treated or the water is getting re-contaminated. The discussion was modified taking your comments into consideration.

A major area requiring attention is the depth of discussion. At present, it is very descriptive of the situation in the area of Ethiopia analysed, but does not infer how this might relate to other regions of the world affected by STH.

This has been taken into account and the discussion has been amended.

What has been learned about risk factors and environment for STH?

Can anything be learned from that about preferred environments for hookworm and Strongyloides (since these were the most common STH recovered)?

How does the data derived from this work compare to other STH studies performed elsewhere in the world or Ethiopia in particular? Were the findings presented a high or low prevalence and intensity of STH infections compared to other regions of Ethiopia?

The discussion was improved as suggested, taken all these very interesting questions into consideration.

The discussion also fails to address any laboratory methodological limitations of the study and avenues for further research.

Would PCR have provided better results?

How does MacMaster compare to the WHO standard method of Kat Katz?

What about hookworm species – as Necator americanus has different levels of environmental resistance to Ancylostoma duodenale, and Ancylostoma ceylanicum has a zoonotic reservoir, would knowing the species of hookworm infecting people by using PCR have provided further information for analysis?

These limitations were taken into consideration and added.

--------------------

Data Presentation

Reviewer #2: (No Response)

Reviewer #3: Please see my comments in the attached .pdf

--------------------

Summary and General Comments

Reviewer #1: Despite the ongoing mass drug administration there is bottle neck about the elimination STH in several countries and this type of assessment done by María Victoria Periago et al. is useful to explore the environmental characteristics and their implications on NTD.

This is very interesting areas of research on Neglected tropical diseases to study the impact of environment on the occurrence or presence of STH under ongoing mass drug administration with the goal to eliminate them in the near future. They came with very important findings showing that open defecation and lack of electricity, being adults are associated with hookworm infection. These findings add new knowledge in the field of NTDs research and will lead to reassess new strategies or improve existing strategies.

However, the authors should report also the few cases of STH before focusing on the hookworm found during their study.

Reviewer #2: This is an interesting study on the spatial distribution of STH in endemic areas in Ethiopia. The study has scientific merit and adds relevant information for the improvement of hookworm control strategies. The article describes the current scenario of eco-epidemiology of STHs, which is the same in different developing countries, in rural or peri-urban populations. This scenario is characterized by: i) permanence of hookworm infections, ii) frequent failure of mass drug administration policies due to re-infections and maintenance of infections in adolescents and adults, iii) permanence of populations practicing open defecation in peridomestic areas, iv) predominance of low parasitic loads. The recognition of this landscape is necessary for the improvement of control policies. Some methodological improvements were pointed out, as well as some aspects related to the interpretation of the data.

Reviewer #3: Please see my comments in the attached .pdf

All the comments and corrections made in the .pdf were revised accordingly.

--------------------

PLOS authors have the option to publish the peer review history of their article (<a href="<https://journals.plos.org/plosntds/s/editorial-and-peer-review-process#loc-peer-review-history>" target="_blank">what does this mean?</a>). If published, this will include your full peer review and any attached files.<br><br>

If you choose “no”, your identity will remain anonymous but your review may still be made public.<br><br>

<b>Do you want your identity to be public for this peer review?</b> For information about this choice, including consent withdrawal, please see our <a href="<https://www.plos.org/privacy-policy>" target="_blank">Privacy Policy</a>.

Reviewer #1: Yes: Housseini Dolo

Reviewer #2: Yes: Filipe Anibal Carvalho Costa

Reviewer #3: Yes: Richard Bradbury
